# The cold responsive mechanism of the paper mulberry: decreased photosynthesis capacity and increased starch accumulation

**DOI:** 10.1186/s12864-015-2047-6

**Published:** 2015-11-05

**Authors:** Xianjun Peng, Linhong Teng, Xueqing Yan, Meiling Zhao, Shihua Shen

**Affiliations:** Key Laboratory of Plant Resources, Institute of Botany, the Chinese Academy of Sciences, Beijing, 100093 China

**Keywords:** Paper mulberry, Transcriptomics, Proteomics, Abiotic stress, Starch synthesis

## Abstract

**Background:**

Most studies on the paper mulberry are mainly focused on the medicated and pharmacology, fiber quality, leaves feed development, little is known about its mechanism of adaptability to abiotic stress. Physiological measurement, transcriptomics and proteomic analysis were employed to understand its response to cold stress in this study.

**Methods:**

The second to fourth fully expanded leaves from up to down were harvested at different stress time points forthe transmission electron microscope (TEM) observation. Physiological characteristics measurement included the relative electrolyte leakage (REL), SOD activity assay, soluble sugar content, and Chlorophyll fluorescence parameter measurement. For screening of differentially expressed genes, the expression level of every transcript in each sample was calculated by quantifying the number of Illumina reads. To identify the differentially expressed protein, leaves of plants under 0, 6, 12, 24, 48 and 72 h cold stress wereharvested for proteomic analysis. Finally, real time PCR was used to verify the DEG results of the RNA-seq and the proteomics data.

**Results:**

Results showed that at the beginning of cold stress, respiratory metabolism was decreased and the transportation and hydrolysis of photosynthetic products was inhibited, leading to an accumulation of starch in the chloroplasts. Total of 5800 unigenes and 38 proteins were affected, including the repressed expression of photosynthesis and the enhanced expression in signal transduction, stress defense pathway as well as secondary metabolism. Although the transcriptional level of a large number of genes has been restored after 12 h, sustained cold stress brought more serious injury to the leaf cells, including the sharp rise of the relative electrolyte leakage, the declined Fv/Fm value, swelled chloroplast and the disintegrated membrane system.

**Conclusion:**

The starch accumulation and the photoinhibition might be the main adaptive mechanism of the paper mulberry responded to cold stress. Most of important, enhancing the transport and hydrolysis of photosynthetic products could be the potential targets for improving the cold tolerance of the paper mulberry.

**Electronic supplementary material:**

The online version of this article (doi:10.1186/s12864-015-2047-6) contains supplementary material, which is available to authorized users.

## Background

The paper mulberry belongs to the family of Moraceae and is naturally distributed in Eastern Asia and pacific countries [1], which has shallow roots with advanced lateral roots and without an obvious taproot. Because of its fast growth and adaptability, the paper mulberry is commonly used for the ecological afforestation and landscape in both sides of highway, mined areas and on barren land [2]. The surface properties of the leaf powder of paper mulberry is suitable for the metal adsorption process, which makes it as an ideal adsorbent to remove heavy metal ions from aqueous solutions [3]. Due to the high contents of flavonoids and other secondary products, the paper mulberry has long been used in Chinese traditional medicine for the treatment of inflammatory disorders [4–8]. Moreover, ethanol extracts of the paper mulberry can significantly interfere with the development of *Plutella xylostella* population [9]. The average length of a phloem fiber is 7.45 mm, and that of xylem is 0.58 mm, which makes it an excellent material widely used in papermaking [10]. Many research have been carried out on paper making and medicine, while there are relatively few studies on physiological and molecular mechanisms responded to abiotic stress.

Temperature is still the major factor that significantly affects the distribution of the paper mulberry just as other tree plants [11, 12] especially in Northern China, which presents a challenge to the further exploitation of the paper mulberry for human needs. *BpDREB2*, cloned from the paper mulberry, can significantly enhance the freezing tolerance of Arabidopsis without causing growth retardation [13]. The molecular mechanism about cold stress tolerance of the paper mulberry has not been studied, which limits its exploitation.

Studies have shown that the transcriptional, post-transcriptional and post-translational regulations of gene expression are critical for the adaptation of cold stress. Because of lacking of the genome reference, the study of tree functional genomics had once been far behind the model plants and crops [14, 15]. However, in the past decade, the development of next-generation sequencing technology has accelerated the process of tree functional genomics research [16]. The deciphering of tree genome, including *Populus trichocarpa*, *Jatropha curcas*, *Prunus mume* and *Morus notabilis*, make the study of stress responsive mechanisms of trees shift from a single gene to the global genomics, transcriptomics and proteomics. For example, when response to cold stress, total of 21 miRNAs are down-regulated and 9 are up-regulated in *Populus tomentosa*, which demonstrate that *Populus* miRNAs play critical roles in the cold stress response [17]. A substantial number of ESTs encoding transcription factors including CBF and high redundancy of genes are identified to be involved in cell protection, dehydrative stress and sugar metabolism from leaves of a cold-acclimated *Eucalyptus gunnii* [18]. Total of 1,770 differentially expressed transcripts have been identified from *Camellia sinensis* during cold acclimation, of which 1,168 are up-regulated and 602 down-regulated, including a group of cold sensor or signal transduction genes, cold-responsive transcription factor genes, plasma membrane stabilization related genes, osmosensing-responsive genes, and detoxification enzyme genes [19]. Additionally, most studies have been carried out on model plants Arabidopsis and rice because of their existed large protein sequence database [20–23] and only few studies has been performed in woody plants. Similar to herbaceous plants, poplar exposed to 4 °C accumulated proteins related to ROS scavenge, such as ascorbate peroxidase, thioredoxin and peroxiredoxin [24]. Moreover, the cold treatment on poplar reduced the Fv/Fm ratio, affected the methionine pathway, as well as HSPs and proteins with known membrane-stabilizing properties [25]. For peach bark tissue exposed to 5 °C, the increased proteins are involved in defense response, lignin metabolism, glycolysis and protein metabolism while decreased proteins belong to cytoskeletal organization, defense response and photosynthesis pathway [26]. Proteomics on *Picea obovata* reveal increased dehydrins, HSP70s, ATPases, lipocalin, cyclophilins, glycine-rich protein and psbP, and decreased malate dehydrogenase, glyceraldehyde-3-phosphatedehydrogenase, methionine sulfoxide reductase and RuBisCO activase [27].

In this study, we tried to explore the cold stress response mechanism by combining the physiological, transcriptomics and proteomic approaches applied on the paper mulberry seedlings under different stress time. Monitoring the dynamic response process under cold stress provided us the opportunity to discover the candidate gene and the potential pathway in the paper mulberry response to cold stress.

## Results

### Ultrastructure and physiological changes under cold stress

**Fig. 1:**
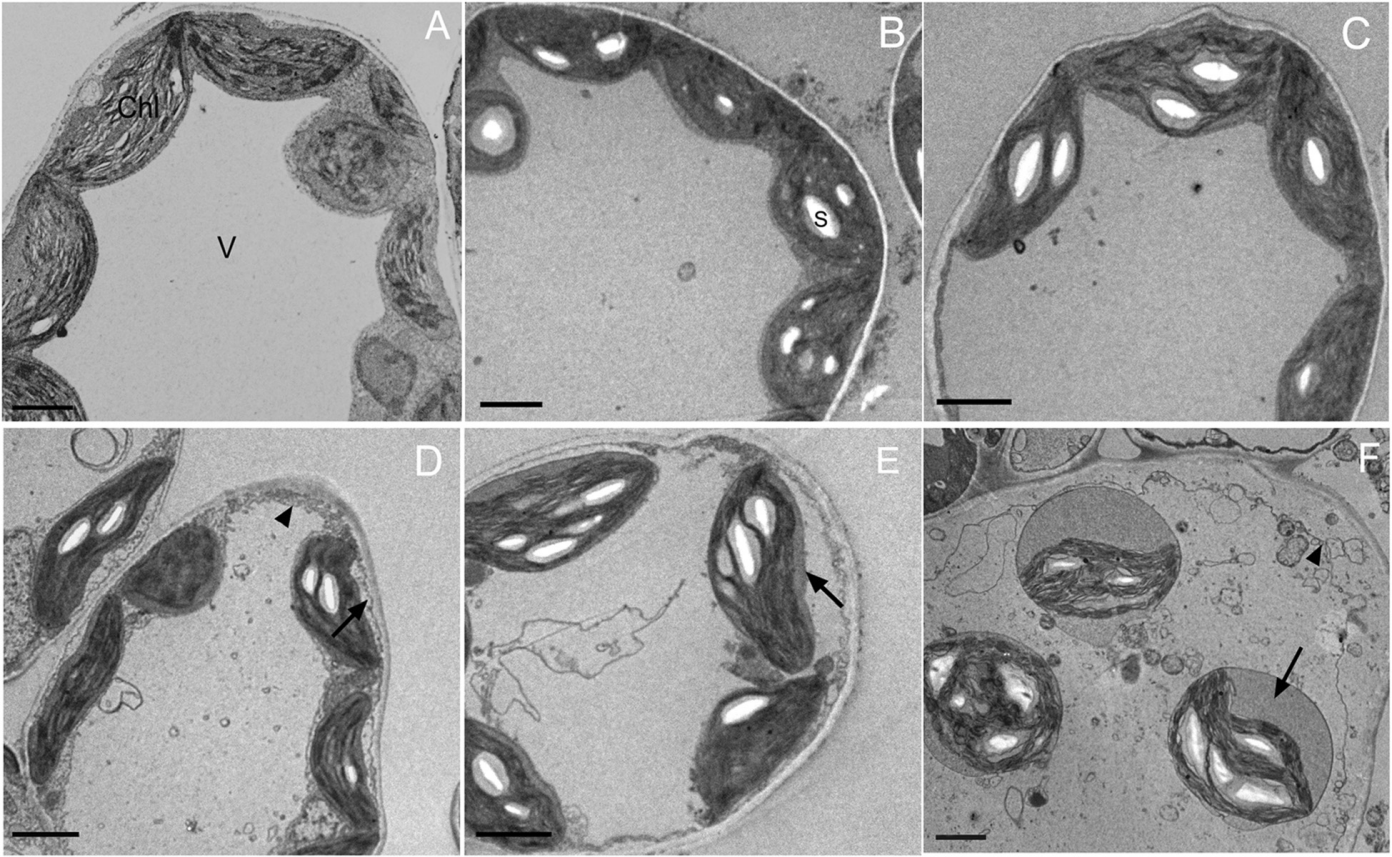
Electron micrographs of paper mulberry leaf cells from control and treated plants. **a** Cells from control plant (0 h); **b** 3 h at 4 °C, showing early stage, chloroplasts with starch grains were noted; **c** 6 h at 4 °C, starch grains were present in many; **d** 12 h at 4 °C, showing invagination of the plasma membrane (arrow) accompanied with amounts of membrane vesicles (arrowhead), note the deformation of chloroplast; **e** 24 h at 4 °C, chloroplast envelope membrane swell (arrow); **f** 48 h at 4 °C, invagination of the plasma membrane and protrusion of vesicles into the vacuoles were noted (arrowhead), swelling continued until plastids were round (arrow). Abbreviations in the figures: V: vacuole, Chl: chloroplast, S: starch grain. Bar = 2 μm

The seedlings of paper mulberry did not display the visible morphological changes within 24 h under cold stress, while with prolonged treatment from 48 h to 72 h, the leaves margin began to wilt. To obtain the ultrastructure changes of the paper mulberry leaf during cold stress, the treated leaves were made into ultrathin slices for TEM observation. The ultrastructure changes of the leaf cell under treatment were shown in Fig. 1a–e.

During the cold stress, the cell membrane showed no obvious change before 12 h (Fig. 1a–c). After 12 h of cold stress, plasma membrane invagination was observed and membrane vesicles were frequently occurred in the vicinity of plasma membrane (Fig. 1d–f). There was obviously damage of the plasma membrane and the tonoplast after cold stress for 24 h. At the 48 h, the tonoplast has been thoroughly ruptured.

At the early stage of cold stress, the chloroplast envelope was well defined and has no significant changes (Fig. 1a–c). After 12 h exposed to cold stress, the chloroplast shape gradually changed from the original shuttle type to the expanding spindle or oblong round. Meanwhile, there was serious damage occurred to the chloroplast membrane structure with low level stacked thylakoid and without the typical grana (Fig. 1d–f).

Another significant change was accumulation of starch grains in chloroplasts throughout the cold period. After 3 h of exposure to cold, cells became enriched with starch grains (Fig. 1b and c). Volume and number of starch grains increased with the prolonged treatment (Fig. 1b–e). Large amounts of starch grains appeared in chloroplasts, almost filling up them (Fig. 1f).

**Fig. 2:**
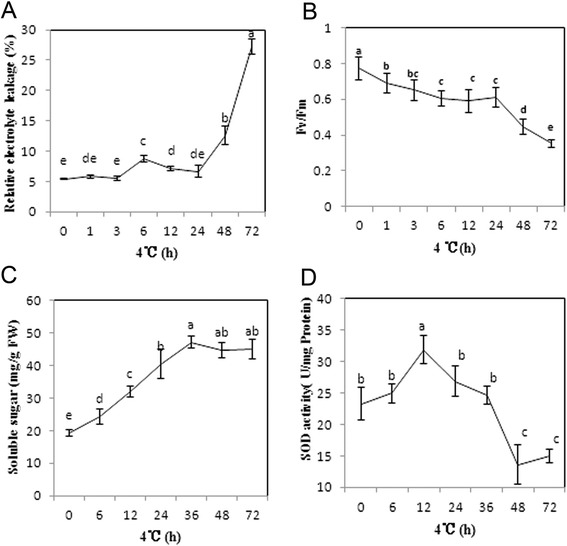
Changes of four physiological traits in leaves of paper mulberry under cold stress. **a** REL. **b** Maximum photochemical efficiency Fv/Fm. **c** Soluble sugar content. **d** SOD activity. Values are presented as mean ± SD of three biological replicates. Duncan’s test (*p* < 0.05) was used for data statistics at different time points and letters a, b, c, d, and e indicated statistical significance

The damage on plasma membrane could also be detected by measurement of REL (relative electrolyte leakage), an indicator of membrane integrity. Impairment of membrane led to leakage of ions from cytosol through cell membranes. The changes in REL were monitored over 72 h cold stresses (Fig. 2a). Under the normal condition (Clonal plantlets were cultured at 26 °C), the REL of the paper mulberry is 5.4 %. It increased moderately at the first 6 h compared with the control, followed by a slightly decrease up to 24 h. After that, the REL increased drastically to 12.57 % at 48 h, and then reached a maximum of 27.19 % at 72 h after cold stress.

Fv/Fm ratio represents the maximum quantum yield of the primary photochemical reaction of PSII. It is an indicator of the functional state of the photosynthetic apparatus. The initial value of Fv/Fm was 0.77 (Fig. 2b). Then there was a slight decrease of it before 24 h, when the value was 0.61. After that, this value decreased rapidly, reduced by 50 % at 72 h.

Plant responses to cold stress are often related to the accumulation of soluble sugar, which may counteract the osmotic stress caused by cold. In this study, cold induced a significant increase in the concentrations of soluble sugar (Fig. 2c), from 19.3 at control to 40.5 mg/g fresh weight at 24 h, and reached the vertex 47.16 mg/g at 36 h.

To explore the plants defense response when exposed to cold stress, the activity of SOD, one ROS scavenging enzyme was measured (Fig. 2d). There was an increase in SOD activity and it reached the maximum of 32 U/mg proteins at 12 h. After 12 h of cold stress, the SOD activity decreased and became lower than that of control after 48 h.

### Transcriptomics of the paper mulberry under cold stress

After stringent quality checks and data cleaning, we obtained 12, 992, 767, 550 raw reads containing a total of 9.164 G nucleotides. The data quality and the assembly results were shown in Table 1. Prior to annotation and differential expression analysis, sequencing saturation was assessed to confirm that enough sequencing data had been obtained for further analysis. The results indicated that the sequencing data of five samples were sufficient for expression analysis (data not shown).

**Table 1:** Summary for the sequencing outcomes and the results of transcriptome assembly

Sample		0 h	2 h	6 h	12 h	24 h
Clean data (Gbp)		1.635	1.875	1.846	1.856	1.952
Percentage of total data (%)		95.86	96.13	95.4	96.43	96
GC (%)		49.22	48.86	49.00	47.61	47.69
Q20 (%)		97.77	97.84	97.61	97.93	97.87
Contigs	Count	690,116	673,180	776,412	687,076	781,156
	N50 Length (bp)	100	139	109	159	150
	Mean Length (bp)	91	103	97	109	106
Transcripts	Count	48841	53,115	51,823	75,284	101,027
	N50 Length (bp)	1190	1623	1432	1891	2028
	Mean Length (bp)	825	1066	944	1234	1343
Unigenes	Count	27,048	30,290	30,655	33,455	36,690
	N50 Length (bp)	1078	1504	1357	1607	1566
	Mean Length (bp)	737	915	856	916	876
	Total Length (Mbp)	19.80	27.71	26.24	30.64	32.13

**Fig. 3:**
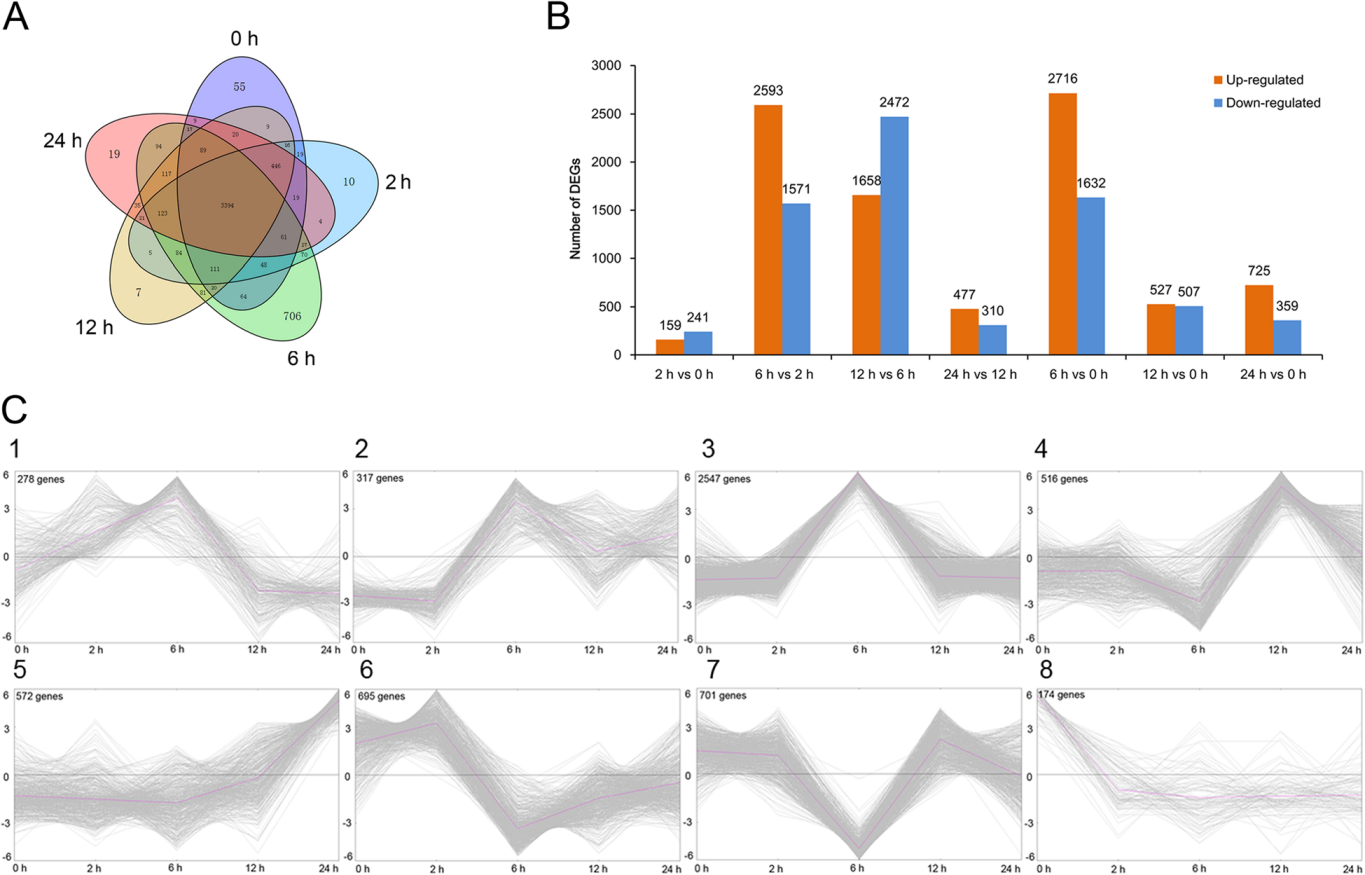
Differentially expressed unigenes in each sample. **a** Venn diagram of differentially expressed unigenes distributed in every sample. The time was represented the sample that were treated under cold stress. The expression pattern and the statistics of the differentially expressed genes in every pairwise comparison. **b** The up-regulated and down-regulated DEGs in every pairwise comparison. Taken pairwise comparison of 2 h vs 0 h as an example, up-regulated was referred to the expression was higher than that after 2 h of cold stress treatment while down-regulated referred to the expression was lower than that after 2 h of cold stress treatment. **c** The expression profile of all the 5800 DEGs during the cold stress. According to their expression pattern, all of them could be divided into 8 groups by using the Mev 4.0 software

To identify DEGs accurately, we dropped off all unigenes with the maximal RPKM < 5 in all sequencing libraries before DEG analysis. By applying screening thresholds of 3 fold changes and FDR ≤0.01, a total of 5,800 DEGs were detected to involve in cold stress. The number of DEGs expressed in every comparison was shown in Fig. 3a. Compared with the untreated leaves, a total of 4,348 genes were differentially expressed after 6 h of cold stress treatment, of which 2,716 were up-regulated and 1,632 down-regulated DEGs. Meanwhile, there were 4,164 DEGs between 6 h and 2 h after cold stress treatment (Fig. 3b). After 12 h of cold stress, the number of DEGs (1034) decreased dramatically compared with the untreated leaves. However, when comparing gene expression at 12 h of cold exposure to 6 h, there was a significant increase in the number of down-regulated genes (2,472), which is more than the number of up-regulated genes (1,658). According to their expression profiles, the 5,800 DEGs can be clustered into 8 groups (Fig. 3c), of which group 3 contained the most DEGs that induced by cold stress at the 6 h, including 2,547 genes and representing the 43.91 % of the total differentially expressed DEGs.

**Fig. 4:**
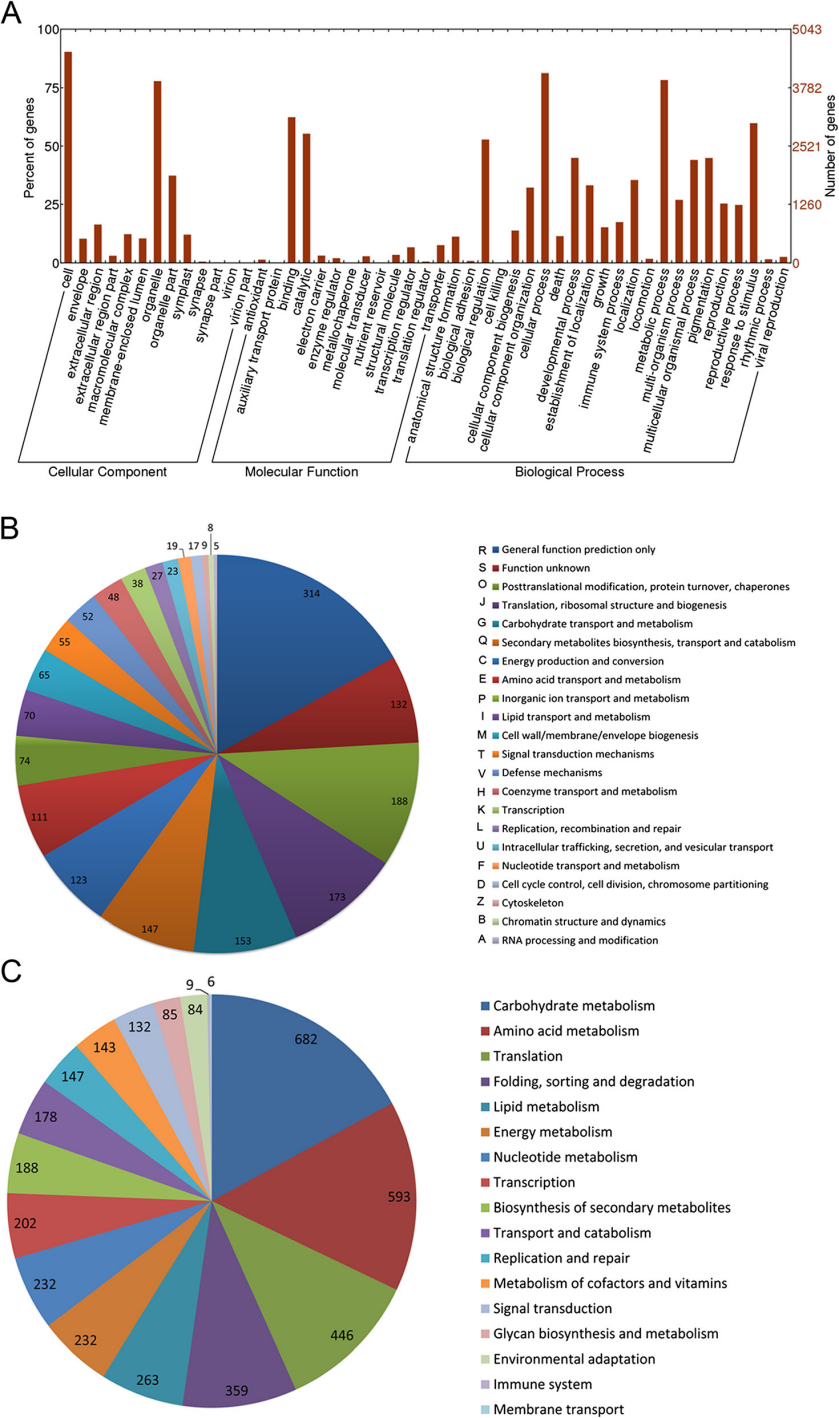
Annotation and functional classification of the DEGs. **a** Histogram of Gene Ontology (GO) classifications of the DEGs in paper mulberry under cold stress. Total 5043 DEGs could be annotated by GO and they mainly distributed in metabolic process, response to stimulus, catalytic, binding and organelle belonged to Biological process, Cellular components and Molecular function, respectively. **b** The number of DEGs in each COG functional classification group. A total of 1,851 DEGs had been annotated by COG. Except the function prediction only and function unkown, group O (Posttranslational modification, protein turnover, and chaperones), J (Translation, ribosomal structure and biogenesis), G (carbohydrate metabolism), C (energy production), E (amino metabolism), P (inorganic ion transport and metabolism), I (lipid metabolism) and Q (secondary metabolism) took up more than half of the total DEGs annotated in COG. **c** The statistics analysis of DEGs mapped to KEGG pathways

Results of annotation and functional classification of the DEGs suggested that total of 5,043 DEGs could be annotated by GO annotation (Fig. 4a and Additional file 1) and made up 86.95 % of the total DEGs. In the cellular component, total 523 DEGs were identified as envelope term. In the molecular function, 848 DEGs, accounted for 16.8 % of the total DEGs, was related to the oxidoreductase activity. In the biological process, 227 DEGs were termed as hormone metabolic process and 1,029 DEGs were involved in the secondary metabolic process. Most important, there were 2,311 DEGs were response to stress under the term of response to stimulus, including biotic stress (1,244) and abiotic stress (1,691). A total of 1,851 DEGs had been annotated by COG (Fig. 4b). Among of these, there were 153, 123, 70 and 147 DEGs were involved in carbohydrate metabolism, energy production, lipid metabolism and secondary metabolism, respectively. In addition, there were 1,122 DEGs, accounting for the 19.36 % of the total DEGs, mapped to the 119 KEGG pathways (Fig. 4c). The majority DEGs distributed in each KEGG pathway was similar to the annotation results of GO and COG.

**Fig. 5:**
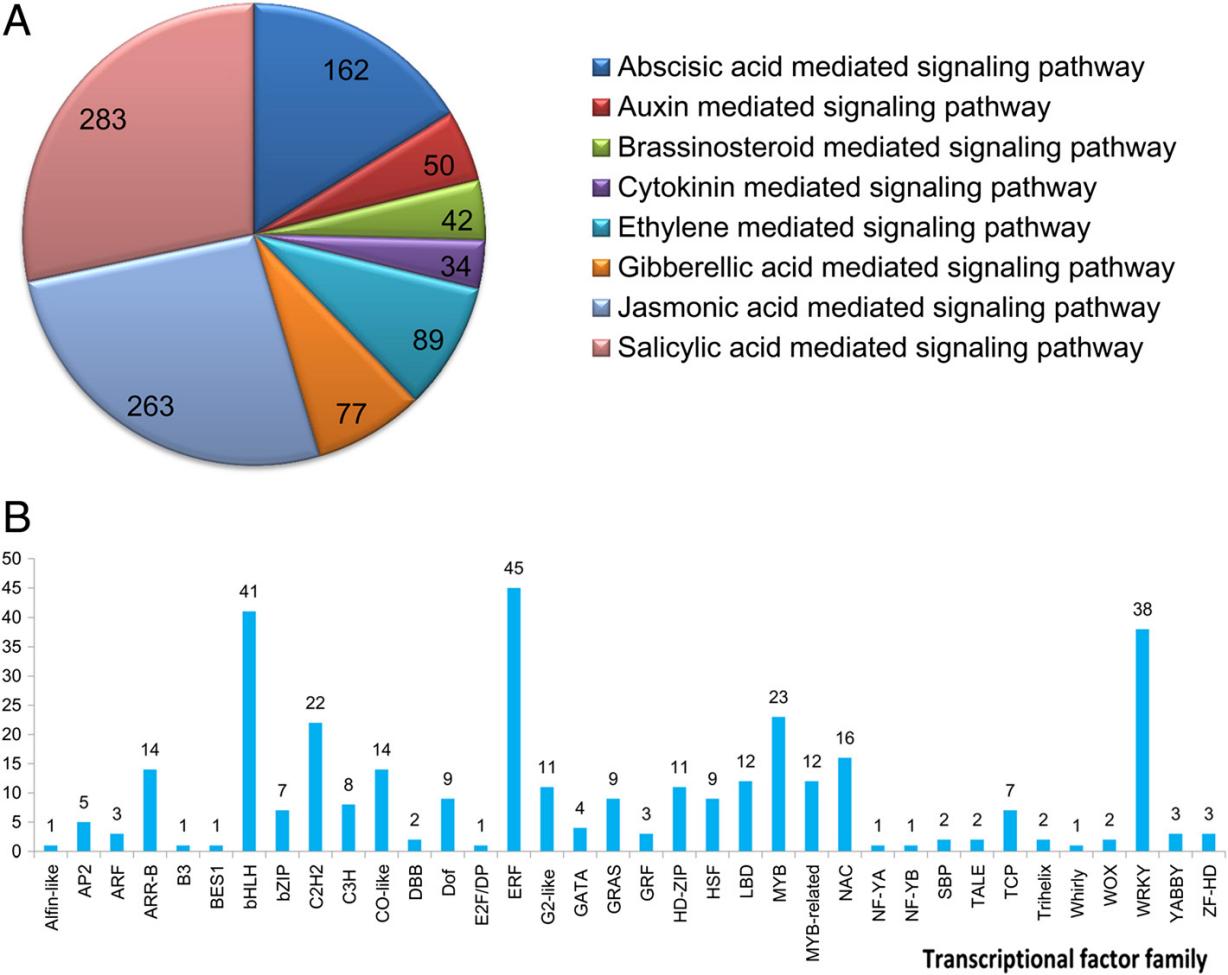
The number of DEGs related to phytohormones signaling pathway and transcriptional factor. **a** Statistics of DEGs involved in the phytohormones signaling pathway. **b** No. of differentially expressed TFs in each family

The signal transduction pathway plays a pivotal role in the response to the stress of low temperatures and cold stress-induced second messenger can be decoded by different pathways, including Ca^2+^, ROS, Receptor-like protein kinases and other signal pathways (Additional files 2, 3 and 4). Phytohormones, such as ABA (abscisic acid), auxin, BR (brassinosteroid ), CK (cytokinin), ETH (ethylene), GA (gibberellic acid), JA (jasmonates) and SA (salicylic acid) are related to the cold responses positively or negatively. They have been shown to play an important role in mediating ROS and other cold stress signals. In our study, there were 162, 50, 42, 34, 89, 77, 263 and 283 DEGs related to ABA, auxin, BR, CK, ETH, GA, JA and SA mediated signaling pathway in the GO annotation result, respectively (Fig. 5a).

One of the major advances in the past decade of cold tolerance research is the discovery of the cold stress related TFs (transcription factors). In the paper mulberry, total 346 TFs belonged to 35 families were responsive to cold stress (Fig. 5b), including 48 *ERF*s, 41 *bHLH*s, 22 *C2H2*s, 23 *MYB*s, 16 *NAC*s, 38 *WRKY*s and so on. Additionally, there were 14 *CO-like*s, 11 *G2-like*s, 9 *GRAS*s, 3 *GRF*s, 12 *LBD*s, 2*WOX*s and 3*YABBY*s, which have been have been characterized primarily for their participation in the regulation of plant development and growth, responded to cold stress in the paper mulberry. The cold stress responded TFs accounted for the 25.9 % of the total TFs of this tree [28].

Photosynthesis and chloroplasts are highly sensitive to temperatures. Low temperatures usually lead to the inhibition of sucrose synthesis and photosynthesis. There were 472 DEGs directly involved in the photosynthesis and 393 of which exhibited the suppressed expression at 6 h after cold stress (Additional file 5), including DEGs encoding ATP synthase gamma chain, Carbonic anhydrase, Chlorophyll a-b binding protein, Oxygen-evolving enhancer protein, Photosystem I reaction center subunit, Photosystem II reaction center protein, Ribulose-1,5 bisphosphate carboxylase/oxygenase large subunit, Ribulose bisphosphate carboxylase small chain and Thylakoid lumenal protein, etc. Detailed information of photosynthesis pathway in KEGG database also indicated that photosystems I and II (PSI and PSII) were seriously influenced by cold stress (Additional file 6). In addition, the genes encoding chloroplastic ribosomal protein were down-regulated at 6 h of cold stress while the genes encoding cytoplasmic ribosomal protein showed the contrary expression trait (Additional file 7). More important, a total of 47 DEGs for pentatricopeptide repeat-containing protein (PPR) which playing constitutive and essential roles in and chloroplasts development were responsive to cold stress and most of them were seriously repressed (Additional file 8A).

Total 153 DEGs were clustered in the “carbohydrate transport and metabolism” in the COG analysis result and 682 DEGs mapping in KEGG database belonged to the “carbohydrate metabolism” pathway (Fig. 4b and c). As showed in Additional files 8B and 9, many DEGs encoding Glycogen phosphorylase, Sucrose-phosphate synthase, Alpha, alpha-trehalose-phosphate-synthase, 1, 4-alpha-glucan branching enzyme, Starch synthase, Glucose-1-phosphate adenylyltransferase and other starch synthesis key enzymes were up-regulated at the 6 of cold stress. However, the DEGs for the glucose synthesis key enzymes were down-regulated, including UDP-glucose-6-dehydrogenase, Alpha-glucosidase, Beta-fructofuranosidase and Beta-glucosidase.

Cold exposure also can induce distinct changes in membrane composition. In our DGE analysis, total 73 genes responded to cold stress, encoding 3-ketoacyl-CoA synthase (10), 3-oxo-Delta (4, 5) -steroid 5-beta-reductase (4), GDSL esterase/lipase (35), Omega-6 fatty acid desaturase (3), Phospholipase (11) and other lipid metabolism related proteins (Additional file 10). Cold stress plays important impact on the cell wall content and component. In our study, a large number of DEGs were related to cell wall component synthesis, including cell wall-associated hydrolase, cellulose synthase, extensin, expansin, glycine-rich cell wall structural protein, leucine-rich repeat extensin-like protein, pectinesterase/pectinesterase inhibitor, pectate lyase, polygalacturonase and vegetative cell wall protein, most of them exhibited the cold stress induced expression characteristics (Additional file 11).

Many secondary metabolites found in plants have the extensively role in defense against biotic and abiotic stress. The paper mulberry is enriched in phenylpropanoid, alkaloid and flavonoid and so on. There were 78 DEGs encoding cytochrome P450s which were involved in the regulation of secondary metabolism (Additional file 12). ABC transporters largely contribute to membrane transport of endogenous secondary metabolites in the plant body. A total of 16 up-regulated and 16 down-regulated ABC transporter genes were identified in our study (Additional file 13).

The composition of lignin and the amount produced are known to be regulated by environmental factors. In the paper mulberry, many DEGs were annotated to the phenylpropanoid metabolism pathway as well as the lignin synthesis (Additional files 14 and 15). Additionally, there were 51 DEGs were related to flavonoid synthesis, such as chalcone--flavonone isomerase (6), chalcone synthase (11), isoflavone reductase (5), isoflavone 2&apos;-hydroxylase (7), UDP-glucose flavonoid 3-o-glucosyltransferase (12), etc. (Additional file 16). Most of them showed the transient up-regulation at 6 h of cold stress. As for alkaloid metabolism, many DEGs realted to Reticulon-like protein (4), Reticuline oxidase-like protein (4), (S)-N-methylcoclaurine 3&apos;-hydroxylase isozyme (12) and Vinorine synthase (5) (Additional file 17) also had the induced expression.

Cross talk often exists among cold stress and other biotic or abiotic stress. In the paper mulberry, a total of 82 DEGs encoding disease resistance response protein responded to cold stress (Additional file 18). Besides, the expression of DEGs encoding Subtilisin-like protease (16), pathogenesis-related protein (8), pleiotropic drug resistance (16) protein, TMV resistance protein (15) and MLP-like protein (14) and Retrovirus-related Pol polyprotein from transposon (12) were also affected by cold stress (Additional file 19). There were still a large number of unknown gene exhibited the cold response traits in the paper mulberry.

### Proteomics analysis

About 600 protein spots were reproducibly detected within each gel. Statistical analysis revealed 38 protein spots to be significantly different (*p* < 0.05) at least at one time point when compared to the control, of which 9 spots were qualitatively and 29 spots were quantitatively different (Additional file 20). The mean value, SD and CV of each differential spot were calculated (Additional file 21). After analyzed by MALDI-TOF MS, 34 protein spots were identified with database searching and categorized into seven functional groups, namely anti-oxidation and defense, calvin cycle, citrate cycle, glycolysis, photosynthesis, protein processing and transcription and translation (Table 2). Five of these proteins, *i.e.*, glyceraldehyde-3-phosphatedehydrogenase (GAPDH, spot 12), elongation factor Tu (EF-Tu, spot 23), plastid lipid-associated protein (PAP, spot 24), chlorophyll a/b binding protein (CAB, spot 27) and peroxiredoxin (Prx, spot 34) were newly accumulated whereas four proteins, including RuBisCO LSU (spot 2), methionine sulfoxide reductase (MSR, spot 28), prefold subunit 2 (spot 31), and oxygen-evolving complex protein (OEC, spot 33) disappeared after stress. The other 18 spots (spots 1, 3–6, 8–11, 13–16, 19, 25, 35–37) showed increased abundance and 7 spots (spots 7, 18, 20–22, 30, 38) showed decreased abundance. Protein species involved in antioxidant defense system, photosynthesis, transcription or translation, and energy metabolism were most abundant. The defense related proteins, i.e., glutathione reductase (GR, spot 6), Prx (spot 35), HSP70 (spot 1), and two proteasome subunits (spot 13, 25) were up-regulated. Interestingly, three of the defense proteins reduced during the stress, including ascorbate peroxidase (APX, spot 21), thioredoxin (Trx, spot 38) and HSP90 (spot 18). Proteins related to photosynthesis were widely affected by low temperature, including up-regulated RuBisCO LSU (spot 4), RuBisCO binding protein (spot 3), RuBisCO activase (RA, spot 10) and down-regulated RuBisCO LSU (spot 7), psbP (spot 20), CAB (spot 30). Another group was transcription and translation related proteins, including up-regulated EF-Tu (spot 11), RNA-binding proteins (RBPs, spot 14, 3 6) and ribonucleoprotein (RNP, spot 19). The EF-Tu protein increased at 12 h, indicating the accelerated protein synthesis. The two RBPs increased during the initial stage, and decreased at 72 h. RNP increased at 24 h. The proteins involved in energy metabolism increased before 48 h, including enolase (spot 5), fumarase (spot 8), malate dehydrogenase (MDH, spot 15), and inorganic pyrophosphatase (inPPA, spot 16), indicating the enhanced energy production. Besides, protein TIC 62 (spot 9), participating in protein transportation, accumulated under cold stress. Nucleoside diphosphate kinase (spot 37) increased at 24 h.

**Table 2:** Differential protein species in paper mulberry leaves during cold treatment identified by MALDI-TOF/TOF MS

Spot no.	Expression pattern 0, 6, 12, 24, 48, 72 (h)	Accession no.	Protein name	Organism	L^a^	Theor. Mr (kDa)/p*I*	Exp. Mr (kDa)/p*I*	Score	SC^b^(%)	NP^c^
Anti-oxidation and defense										
6		BAD27393	Glutathione reductase	*Zinnia violacea*	CP	53.4/6.49	53/5.98	127	4	1
21		EXC33221	L-ascorbate peroxidase	*Morus notabilis*	CP	27.4/5.43	27/6.16	242	11	3
28		XP_004501840	Methionine sulfoxide reductase	*Cicer arietinum*	-	22.3/5.38	25/6.69	306	22	4
34		EXB55011	Peroxiredoxin	*Morus notabilis*	-	22.6/8.91	20/6.33	117	5	1
35		ABK22037	Peroxiredoxin	*Picea sitchensis*	C	25.7/9.11	16/4.67	187	12	2
38		EXB51369	Thioredoxin M4	*Morus notabilis*	C	20.2/9.14	14/4.50	135	10	4
Calvin cycle										
10		EXC10712	Rubisco activase	*Morus notabilis*	C	52.1/6.39	43/5.12	476	14	3
2		AAN63283	Rubisco LSU	*Broussonetia papyrifera*	C	51.5/5.83	65/4.91	264	12	2
4		AAN63283	Rubisco LSU	*Broussonetia papyrifera*	C	51.5/5.83	59/4.89	333	15	3
7		AAN63283	Rubisco LSU	*Broussonetia papyrifera*	C	51.5/5.83	51/6.41	598	20	8
3		P34794	RuBisCO LSU-binding protein	*Brassica napus*	C	61.7/5.14	61/4.85	144	4	1
Citrate cycle										
8		AAB39989	Fumarase	*Arabidopsis thaliana*	M	53.4/7.98	50/6.99	239	8	3
15		EXC01795	Malate dehydrogenase	*Morus notabilis*	M	35.9/6.11	37/6.33	279	18	4
37		EXC35650	Nucleoside diphosphate kinase group1	*Morus notabilis*	-	16.3/6.84	15/7.00	338	32	4
16		EXB51441	Solute inorganic pyrophosphatase	*Morus notabilis*	-	33.4/6.61	33/5.01	380	22	3
Glycolysis										
5		XP_002267091	Enolase	*Vitis vinifera*	CP	48.3/6.17	58/6.06	230	8	1
12		EXC04020	GAPDH	*Morus notabilis*	CP	38.4/7.68	39/7.0	179	12	3
Photosynthesis										
20		EXC20796	PsbP domain-containing protein 5	*Morus notabilis*	C	30.5/7.72	28/6.94	162	11	2
33		XP_002300858	oxygen-evolving complex protein 2	*Populus trichocarpa*	C	28.3/7.68	19/5.63	105	10	1
22		EXB93720	Plastid lipid-associated protein 6	*Morus notabilis*	C	30.7/8.53	27/4.96	175	18	1
24		EXB93720	Plastid lipid-associated protein 6	*Morus notabilis*	C	30.7/8.53	26/5.16	155	18	2
27		EXC34900	Chlorophyll a/b binding protein	*Morus notabilis*	C	28.5/5.46	25/4.79	92	9	1
30		EXB80094	Chlorophyll a/b binding protein	*Morus notabilis*	C	26.7/6.10	21/5.56	489	15	5
Protein processing										
1		XP_007033710	heat shock protein 70 isoform 1	*Theobroma cacao*	C	71.6/5.67	70/4.88	1094	18	7
18		EXB97663	Heat shock protein 90	*Morus notabilis*	-	83.7/4.82	30/4.54	780	11	7
31		ACG38358	Prefoldin subunit 2	*Zea mays*	-	16.3/5.84	20/5.28	136	19	1
13		XP_007223366	Proteasome subunit	*Prunus persica*	-	40.4/8.10	37/5.00	227	13	3
25		EXC32303	Proteasome subunit	*Morus notabilis*	-	33.7/8.83	26/6.20	713	32	8
9		EXC10716	Protein TIC 62	*Morus notabilis*	C	51.1/7.68	43/4.90	153-	3	1
Transcription and Translation										
23		EXB94487	Elongation factor Tu	*Morus notabilis*	C	52.2/6.30	38/5.09	108	3	1
11		EXB94487	Elongation factor Tu	*Morus notabilis*	C	52.2/6.30	39/5.12	397	15	4
14		EXB39373	mRNA-binding protein	*Morus notabilis*	C	44.5/7.11	38/6.41	292	12	4
19		EXC31412	Ribonucleoprotein	*Morus notabilis*	C	38.1/4.68	29/4.52	118	9	1
36		AEW08900	RNA binding protein	*Pinus taeda*	C	5.3/4.89	15/4.93	49	19	1

^a^Subcellular location; C: chloroplast; CP: cytoplasm; M: mitochondria; −: no site information

^b^Protein sequence coverage

^c^Number of matched peptides with significant scores identified by MALDI-TOF/TOF MS

According to multivariate analysis with a factor reduction to the 38 differential spots, a general picture of the main variation and of interrelation between spots had been depicted. The first 18 and 17 PCs accounted for 100 % of the variability of gels and differential spots, respectively (Additional file 22). By employing PC1 and PC2 in 2-D plot, gels from the same cold treatment clustered together and could be effectively separated from the other (Additional file 23A). Briefly, samples of 6 h were more closely related to the control. Samples of 12 h treatment formed a cluster, and the other three treated samples were nearer to each other. From this 2-D plot, we can infer that protein abundance changes in response to cold mainly occurred after 12 h and the changes are more similar from 24 h to 72 h. From the 2-D plot projected by protein spots (Additional file 23B), it can be seen that the responsive proteins largely followed into five groups.

### QPCR analysis

As shown in Additional file 24, the accurate of RNA-seq was confirmed by q-PCR. However, some of the proteomics change patterns were not identical with the gene expression profile. The gene expression patterns of 23 genes were similar with that of proteomics data, including all of the genes involved in Calvin cycle and Citrate cycle. The gene expression patterns of other 11 genes were inconsistent with that of proteomics accumulation profile, distributed in Anti-oxidation and defense (3), Glycolysis (1), Photosynthesis (2), Protein processing (2) and Transcription and Translation (3). Of these 11 genes, there was no transcriptional expression difference of T2-33383 (GAPDH), T7-22889 (Prf), T7-29172 (EF-Tu) and T5-14977 (PAP) whereas their protein was accumulated or decreased under cold stress. The inconsistent changes between the transcriptional and proteomic data suggested there were more sophisticated regulation and modification in the post-transcription and the post-translation.

## Discussion

**Fig. 6:**
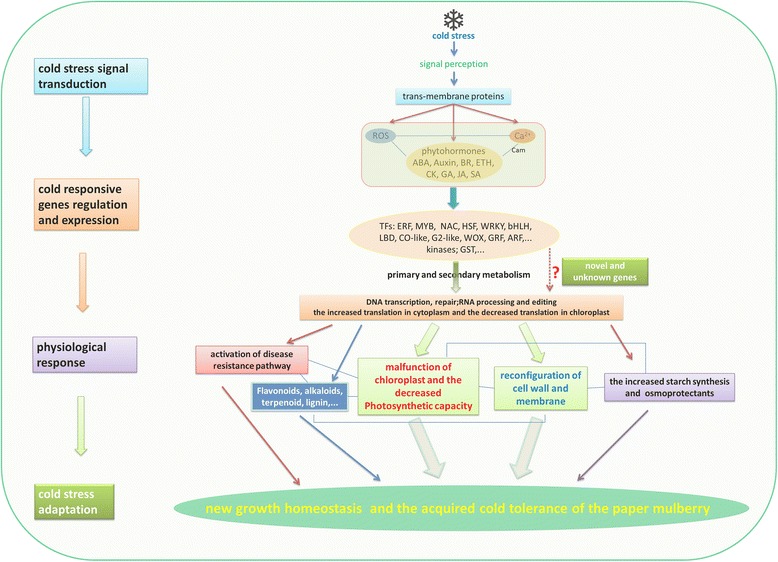
The supposed responsive mechanism of the paper mulberry exposed to cold stress. After signal reception, stress-activated Ca^2+^ signaling, ROS and hormone signaling modulate the expression of stress-responsive transcription factors, kinases, GST and so on. And then activated the expression of genes related to primary and secondary metabolism. The physiological changes mainly included chloroplast malfunction, starch accumulation, cell wall and membrane reconfiguration. Taken together, they endowed the paper mulberry with the new growth homeostasis under cold stress

The present results of ultrastructure, physiologic, transcriptomic and proteomic investigation of the paper mulberry leaf reveals a complex cellular network affected by the low temperature stress. The network covers a broad metabolic process as illustrated in Fig. 6, including cold stress signal perception and transduction, cold stress responsive genes expression regulation, physiological response and finally the cold stress adaptation. In addition to those associated with osmolytes metabolism, chloroplast development, photosynthesis, cell wall remodeling, lignin metabolism, secondary metabolism, starch metabolism and other biotic or abiotic response pathway are positively or negatively correlated with global response to cold stress.

### The impaired cell membranes and the remodeled cell wall under cold stress

Plants may sense low temperature through changes in the physical properties of membranes, because membrane fluidity is reduced during cold stress [29]. According to our results, the cellular ultrastructure was deeply affected after 12 h cold stress (Fig. 1), just as that plasma membrane invagination was frequently observed [30]. One particular observation was the accumulation of vesicles. The increased vesicularization during cold stress can be an indicator of progressive membrane damage as reported under other stress situations [31]. Impairment of membrane led to leakage of ions from cytosol through cell membranes. The change curve of the REL shown in Fig. 2a indicated that cold stress leading to a slight change of membrane stability from 3 to 24 h and the followed drastically increase elucidated that severe membrane damage had occurred after 24 h.

Transcript levels associated with lipid metabolism genes are generally suppressed by low temperature [32]. However, some evidence derived from *Arabidopsis* shows that a number of lipid catabolism enzymes are activated by a fall in temperature, and this is followed by a rise in the amount of free fatty acids present [33]. Based on our transcriptomics result (Additional file 10), most of lipid metabolic genes were down-regulated while some genes were up-regulated suggesting the slowed lipid metabolism process.

Extensin play an important structural role in the acclimation of cold stress, by increasing rigidity of the cell wall and thereby increasing resistance to collapse caused by freeze-induced dehydration [34]. In our study, a large number of DEGs encoding extensin, expansin as well as other cell wall related proteins were up-regulated (Additional file 11). In addition, the cellulose synthesis was also increased because of many up-regulated cellulose synthase. Therefore, we inferred that, during cold stress, the cell wall of the paper mulberry experienced the confrontation between loosening and rigidifying. Finally, the cell wall architecture of the paper mulberry was reconfigured to adapt to cold stress.

### The defense response of the paper mulberry under cold stress

One biochemical change occurring when plants are subjected to abiotic stress is the accumulation of excessive ROS [35]. During the course of evolution, plants have evolved a series of antioxidants to scavenge excess ROS and maintain a cellular redox balance [36]. SOD is providing one of the first lines of defense against the toxic effects of ROS, catalyze the formation of H_2_O_2_ and O_2_. H_2_O_2_ produced is then scavenged by CAT and a variety of peroxidases. In this work, the SOD activity reached vertex at 12 h, indicating its key role in ROS scavenges (Fig. 2d).

Phytohormones, such as ABA, auxin, BR, CK, ETH, GA, JA and SA have been shown to play an important role in mediating ROS and other cold stress signals [29]. The ABA level increases in response to cold stress and the *CBF* genes can be induced by exogenous ABA. The cold response has been reported to involve both ABA-dependent and independent pathways [37]. Recent studies demonstrate that protection against cold stress-induced oxidative damage involves kinase, calcium and phytohormones. Calcium, ABA, SA and ETH are also shown to enhance the activities of different ROS-scavenging enzymes under cold stress, suggesting the complex and essential roles for these signaling pathways in acquired cold tolerance.

Recent studies have shown that ROS also plays a key role in plants as signal transduction molecules in mediating responses to environmental stresses [38]. ROS sensors could be activated to induce signaling cascades that ultimately impinge on gene expression [39]. Several studies have indicated that HSFs (heat shock transcription Factors) and HSPs (heat shock proteins) are involved in the sensing of ROS [40]. There were 9 HSPs, 9 HSFs and many ERFs responded to cold stress in the paper mulberry (Fig. 5), suggesting their potential roles in the ROS signaling pathway. Further studies are needed to elucidate the cause-and-effect relationships between ROS, phytohormones, calcium and kinase during cold stress.

Besides, cold stress has been shown to enhance the transcript, protein, and activity of different ROS-producing or scavenging enzymes, including APX (ascorbate peroxidase), AOX (ascorbate oxidase), G-6-PD (glucose-6-phosphate dehydrogenase), GST (glutathione S-transferase), GR (glutathione reductase), GP (glutathione peroxidase), MDAR (monodehydroascorbate reductase), respiratory burst oxidase, oxygen-evolving enhancer protein, Peroxidase and so on [38, 39]. Recent studies have emphasized that respiratory burst oxidase homologues (RBOHs) are the key signaling nodes in the ROS gene network. There were 5 RBOHs up-regulated at 6 h after cold stress in the paper mulberry, which implied their important roles in the ROS signal transduction. In addition, total of 35 peroxidases, 9 HSPs, 11 peroxisomal related proteins, 4 G-6-PDs, 4 APXs, 16 GSTs, 1 GP, 1 GR, 1 MDAR and 5 other genes were involved in the ROS signaling pathway in the paper mulberry under cold stress.

Prxs are ubiquitous thioredoxin-or glutaredoxin-dependent peroxidases which destroy peroxides [41] and play a crucial role in the antioxidant defense of plant cells [42]. During cold stress in the paper mulberry, Prxs were accumulated and this increase was not accompanied with its transcript, which may be due to an enhancement of Prx translation.

APX, combined with the effective AsA-GSH cycle functions to prevent the accumulation of toxic H_2_O_2_ in photosynthetic organisms [43]. Similarly, APX showed a declined level in present study. This has also been reported in chickpea under cold stress [20]. Simultaneous up and down regulation in different APX isoenzymes were also detected under cold stress, indicating the functionally different APXs [44, 45]. It has been reported that chlAPX in transgenic tobacco under high light and drought conditions are completely inactivated, while the initial activity of SOD is maintained [46], suggesting that APX is much more susceptible to oxidative stress than some other ROS-scavenging enzymes. Besides, antioxidant enzymes MSR also decreased, which was the same case as poplar [25]. MSR, reducing methionine sulfoxide to methionine, can restore the activity of proteins and plants overexpressing of the gene has a higher resistance to chloroplastic oxidative damage [47]. The change in the abundance of the different antioxidant enzymes exemplified cold induced proteome remodeling.

### Chloroplast structure and photosynthetic rate were seriously affected by cold stress

It has been shown that temperature and light intensity changes trigger an imbalance between the energy absorbed in the light phase of photosynthesis and its consumption in photosynthetic metabolism, and affect the relative redox state of PSII [48]. In *Anthurium,* photosynthesis pathway is significantly enriched after 1 h cold treatment, suggesting a role in early response to low temperature [49]. About 50 % of the differentially changed proteins directly involve in the process of photosynthesis before and during cold acclimation in meadow fescue [50]. In barley, it has been reported that light and photosynthetic activity play the major role in frost resistance under cold conditions [51].

According to the analysis of the DEGs involved in the photosynthesis or chloroplasts, we found that 393 DEGs exhibited the suppressed expression at 6 h after cold stress in the paper mulberry. In addition, the genes encoding chloroplastic ribosomal protein were down-regulated, which suggested the protein translation were decreased in the chloroplast. The decreased efficiency of photosystems could also be explained by a degradation of some photosynthetic proteins, including RuBisCO LSU (spot 2 and 7), CAB (spot 30), OEC (spot 33), psbP (spot 20). PsbP proteins are extrinsic subunits of PSII and function in the stability of the water-oxidizing complex of PSII during cold stress [22]. Plants lacked psbP show a marked decrease in the quantum yield evaluated by chlorophyll fluorescence and are hypersensitive to light [52]. The 8 h of 4 °C treated chickpea had the decreased PsbP [20] while freezing tolerant species *Picea obovata* had the increased one [27]. In the present study, PsbP protein showed a constant decrease with duration of cold treatment and little detection at later time points, suggesting they were hyper sensitive to cold, which might be one potential target for molecular breeding. Another component of electron transfer chain OEC disappeared, also suggesting the disturbed electron transfer. Besides, the association of PAP with lipids involved in the structural stabilization of thylakoid membranes under abiotic stress [53]. Both two PAP spots (22, 24) accumulated, suggesting its protection role on the chloroplast membrane system.

The maximal photochemical efficiency Fv/Fm is inversely proportional to the PS IIdamage [54]. In the present study, this value slightly decreased before 24 h, suggesting a relatively stable photosystem. However, after 24 h stress, there was a sharp decline, with Fv/Fm reduced to 50 % at 72 h. These result supported the fact that the chloroplast development of the paper mulberry leaf was seriously affected by cold stress (Fig. 1). Researches on rice show that the Fv/Fm value is reduced by 75 % after 24 h of 4 °C treatment [55]. For *Arabidopsis*, the value is reduced by 25 % after 5 °C treatment for 24 h and 75 % after treatment for 72 h [56]. Thus we inferred that paper mulberry was not so cold tolerance as the two herbaceous plants though it exhibits more tolerance to the short-term cold stress.

Another remarkable event occurred in chloroplast is the accumulation of starch grains (Fig. 1), which appears to result from an imbalance between photosynthate production and export. Low temperature might cause reversible down-regulation of PSII through the dissipation of excess absorbed energy and damage to reaction center proteins and subsequent inhibition of the photosynthetic capacity, namely photoinhibition [57]. Photoinhibition may generate ROS, which cause destruction of the photosynthetic apparatus and damage of whole cells. To avoid this, plants need to seek balance between light energy absorption and utilization via two main strategies: either by increasing light energy dissipation or by increasing the intensity of light energy consumption [58]. The CAB polypeptides are major constituents of the light harvesting complex of thylakoid membranes [59]. The enhanced level of CAB (spot 27) might function by dissipating the excess amount of energy in the paper mulberry. One possible regulatory mechanism of Chl a/b complexes is the aggregation of energy-dissipating pigments contributing to non-photochemical quenching and protecting the photosynthetic apparatus from damage due to excess light energy [60]. Besides, PSI is known to bind approximately half of the chlorophyll in leaves [61]. These results suggested an imbalance between the capacity for harvesting light energy and the capacity to consume this energy on metabolic activity occurs in the paper mulberry leaves under cold stress.

Many DEGs encoding Glycogen phosphorylase, Sucrose-phosphate synthase, Alpha, alpha-trehalose-phosphate-synthase, 1, 4-alpha-glucan branching enzyme, Starch synthase, Glucose-1-phosphate adenylyltransferase and other starch synthesis key enzymes were up-regulated at the 6 of cold stress. However, the DEGs for the glucose synthesis key enzymes were down-regulated, including UDP-glucose-6-dehydrogenase, Alpha-glucosidase, Beta-fructofuranosidase and Beta-glucosidase. Additionally, plant cells can maintain photostasis under cold by increasing electron sink capacity through up regulation of CO_2_ assimilation and carbon metabolism [62]. The proteomic results in our study showed that some carbon metabolism related enzymes are accumulated (Table 2). Chill-induced loss of RuBisCO activity (RA) plays a vital role in the response of photosynthesis to temperature [63]. RA levels increased during cold treatment enhance CO_2_ fixation. RuBisCO LSU binding protein has a role in preventing the irreversible aggregation or promoting the correct folding of proteins [64]. The increase in binding protein is needed to protect RuBisCO from degradation or allow the assembly of more RuBisCO holoenzymes, suggesting the maintenance of Calvin cycle under low temperature. These supported the result that starch was accumulated when the paper mulberry exposed to cold stress.

Additionally, it has been proposed that up-regulation of catabolic pathways could be observed under cold [65]. There were four enzymes involved in catabolism, including GAPDH, enolase, fumarase and MDH (Table 2). Produced energy by catabolism was required for reinforcing plant resistance to cold stress or compensating the reduced enzyme activity caused by cold. So, this could be accounting for the increase of the soluble sugar at the early stage of cold stress (Fig. 2c). Similarly, starch and soluble sugar of the winter wheat and other herbs accumulate in parallel in early autumn cold acclimation, and when at lower temperature, starch hydrolysis with increased soluble sugar content, suggest that the early accumulation of polysaccharides is used for improving the soluble sugar content in late winter. Besides, these enzymes may also function in another way. Studies on *Arabidopsis* have revealed that enolase of nucleus functioned as a transcriptional repressor of *STZ/ZAT10*, a repressor of *CBF/DREB1* pathway [66], by which enolase indirectly activated CBF regulon. Therefore, enolase plays an important role not only in energy metabolism, but also in regulating cold related gene expression.

fUnder normal condition, starch was of sucrose synthesis and photosynthesis. The decline in the rate of photosynthesis leads to the triose phosphate from the RPP cycle (reductive pentose phosphate cycle) transported for starch synthesis. Meanwhile, the photosynthetic fixation of CO_2_ is very limited under low temperatures, photoinhibition occurs even under relatively low irradiance [67, 68]. During low temperature stress or cold acclimation, spring as well as winter wheat exhibited a decreased abundance of starch and increased levels of sucrose even though the starch and soluble sucrose all accumulate simultaneously at the early stage of cold accumulation [67]. It is well known that sucrose is the free sugar commonly accumulated in response to low temperature and probably results from the differential low-temperature sensitivity of the enzymes of starch, sucrose and fructan metabolism [33]. Starch tests also show that the starch content of *L. lancifolium* leaves increase at the early cold treatment, and fell during subsequent cold stress as it is degraded into soluble sugar, which account for that a subset of starch metabolism enzyme genes display a highly specific up-regulation response to cold stress [69]. Although there are repeated reports demonstrating that chilling injury caused reduced size and number of starch granules [70], care must be taken to avoid confusing chilling injury with cold adaptation. Chilling injury inevitably affects photosynthesis, hampering photosynthate production. While starch accumulation was also found in cold-stressed *Chlamydomonas reinhardtii*, suggesting it is a quick adaptive mechanism as a metabolic switch for storage compound synthesis [71]. Diagrammatic summary of cellular metabolic and regulatory pathways in Arabidopsis shows that photosynthetic light reactions, Calvin cycles, starch synthesis in the chloroplast, the photorespiration in the peroxisome and mitochondrion, sucrose synthesis, glycolysis, cell wall synthesis in the endoplasmic are up-regulated whereas water transport, ethylene synthesis in vacuole and cytosol are down-regulated by light [72] and HY5 is the convergence point of light and multiple hormone signaling pathways [73].

**Fig. 7:**
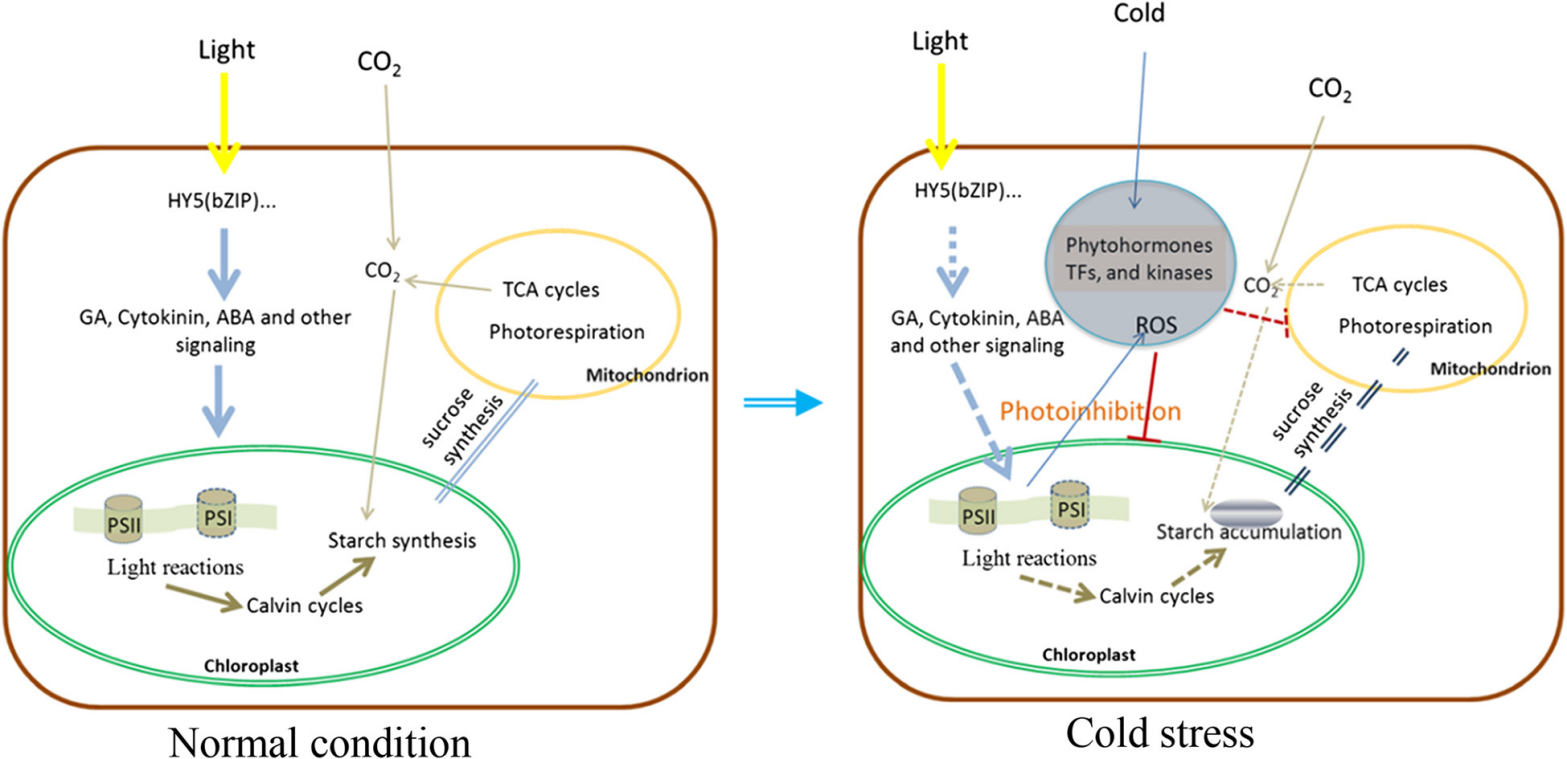
The exhibition of the Physiological process under normal condition and cold stress. Under normal condition, there was a dynamic equilibrium between starch syntheses and transformation into triose phosphate from the photosynthesis product. The sustained cold stress led to the photoinhibition, which affected the synthesis of the sucrose and these conversely led to the accumulation of the starch in the chloroplast. The dashed was used to illustrate the slower physical process and the inverted T-shaped line showed the repressed physical process. Double dotted line showed that the dynamic equilibrium of the triose phosphate transportation and the sucrose synthesis had been broken

Under normal condition, starch was synthesized in the chloroplast and then is catalyzed into triose phosphate which was transported out and used for the synthesis of the sucrose. TCA cycles supply the ATP for these processes. During cold stress, the photorespiration were repressed, which affected the transportation of triose phosphate and the synthesis of the sucrose and these conversely led to the accumulation of the starch in the chloroplast though the synthesis rate of starch were slower caused by the photoinhibition under cold stress (Fig. 7).

## Conclusions

In conclusion, the ultrastructure observation, physiological measurement, transcriptomics and proteomic analysis allowed us to draw a global cold responsive mechanism of the paper mulberry. The evidences in present study suggested the malfunction of chloroplast, the decreased photosynthetic capacity, the accumulated starch and the reconfiguration of cell wall and membrane in the paper mulberry leaf under cold stress. We inferred that the young seedlings of paper mulberry couldn’t resist the long cold stress, which might account for that there was no distribution in Northern China. Our study could serve as a valuable resource for molecular breeding of the paper mulberry to enhance the tolerance and the growth ability of the tree under cold stress.

## Methods

### Plant material, transmission electron microscope (TEM) observation

Clonal plantlets of the paper mulberry, regenerated from the young leaves, were cultured on MS culture media (Murashige and Skoog) at 26°C and a 14/10 h photoperiod (day/night). Three month old plantlets with the same crown size and equal height were chosen and moved into growth chambers for cold stress (4 °C). The mixed sampling strategy has been adopted to eliminate differences between individuals in our study. In addition, the control in this paper is just same one in previous study [1].

The second to fourth fully expanded leaves from up to down were harvested at different time points for the following studies. Thin leave of control and stressed seedlings (0, 3, 6, 12, 24, and 48 h), fixed in 3 % glutaraldehyde in phosphate buffer (0.1 M, pH 7.0–7.2) for 1.5 h at room temperature, were dehydrated to propylene oxide and embedded in Spurr’s epoxy medium. Ultrathin sections (60 nm thick) were stained with uranyl acetate and lead citrate. TEM observations were made on a JEM-1230 TEM with the accelerating voltage of 80 kV (Japan electron optics laboratory co., LTD, Japan).

### Physiological characteristics measurement

The relative electrolyte leakage (REL) assay was performed according to the protocol previously described [45]. The SOD activity assay was carried out according to the reference [74]. Soluble sugar content was measured by Anthrone Colorimetry. Chlorophyll fluorescence parameter was determined in the second fully expanded intact leaves from each plant after 20 min of dark adaptation with an IMAGING-PAM Chlorophyll Fluorometer (Heinz Walz GmbH, Effeltrich, Germany).

### Transcriptomics analysis

fThe libraries were prepared according and 24 h cold stress were used for total RNA isolation. The quality and purity of RNAs were assessed with OD260/230 ratio and RNA integrity number (RIN) by using the NanoDrop 2000 (Thermo Fisher, Waltham, USA ) and the Agilent 2100 Bioanalyzer (Agilent Technologies, Santa Clara, USA), respectively.

The libraries were prepared according to our former study [1]. Raw sequence data were generated by the Illumina pipeline and are available in NCBI’s Short Read Archive (SRA) database under accession number accession number SRP029966. All of the clean reads were pooled together and assembled to form the global transcriptome of the paper mulberry. Besides, to obtain the transcriptome of each sample, raw data was also assembled respectively for each sample (Table 1). The functional annotation of all unigenes and DEGs were annotated as our former study [1].

The expression level of every transcript in each sample was calculated by quantifying the number of Illumina reads that mapped to transcriptome of paper mulberry. The raw gene expression counts were normalized using the RPKM (Reads per kb per million reads) value. For screening of differentially expressed genes, p value corresponds to differentially expressed genes (DEGs) was obtained via a general Chi squared test that was performed by using IDEG6 (http://telethon.bio.unipd.it/bioinfo/IDEG6/). The threshold of p value in multiple tests was checked through manipulating the false discovery rate (FDR) value. Among the five samples, the transcripts with the highest RPKM value more than 5 and the ratio of RPKM between samples of more than 3 (Fold change ≥3) and an FDR ≤0.01 were considered to have significant changes in expression in response to cold stress. The Multiexperiment Viewer (v4.9) was used to make the heat map and expression classification. The COG, GO and KEGG enrichment functional annotations of DEGs were performed using the methods as described above.

### Proteomics analysis

fResults for the control and cold stress samples, 24, 48 and 72 h cold stress were harvested for proteomic analysis. For each protein sample, 300 mg of leaves was used. The protein extraction was performed using a TCA/acetone method [75]. Protein concentration was determined according to Bradford method [76]. The protocols for SDS-PAGE, 2-DE images analysis, differentially expressed protein spots identification were the same as the former study [75]. Three independent protein samples were extracted for each treatment with each sample.

Results for the control and cold stress samples were analyzed for differences using the analysis of variance (ANOVA, *P* < 0.05). Protein spots were selected when a significant variation existed as compared with the control in at least one time point. The isoelectric point and molecular weight of proteins of interest were calculated according to the instruction. Besides, principal component analysis (PCA) was performed, three replicated gels of each treatment were projected on the factor plane to assess the gel repetition, and the differential expressed proteins were projected on the factor plane to view the global spots distribution pattern. Differentially expressed protein spots were clustered with Cluster 3.0 and plotted with Java Treeview software. Protein identification was performed by searching in the non-redundant NCBI database using the MASCOT software package.

### Validation by qPCR

fThe detailed characterization of the GO function the DEG results of the RNA-seq and the proteomics data. RNA used for validation was the same as that for RNA-seq. Gene-specific primers were designed using the Primer 5 program and are listed in Additional file 25. QPCR reaction conditions and volume was performed as described by our former study [1]. The expression level of *actin* gene was used as a control.

## Additional files

Additional file 1:
**The detailed characterization of the GO function enrichment for DEGs.** (XLSX 20 kb) Additional file 2:
**The expression pattern of calcium signaling pathway related DEGs.** (TIFF 804 kb) Additional file 3:
**The expression pattern of DEGs related ROS signaling pathway.** (TIFF 2311 kb) Additional file 4:
**DEGs referred to Receptor-like protein kinases.** (XLSX 20 kb) Additional file 5:
**Heat map of expression profiles of DEGs involved in the photosynthesis.** According to their expression pattern under cold stress, the 472 DEGs were categorized in to two groups, including 393 and 79 DEGs, respectively. Red indicates high expression, black indicates intermediate expression, and green indicates low expression. (TIFF 634 kb) Additional file 6:
**The diagram illustrated for photosynthesis.** (TIFF 1161 kb) Additional file 7:
**The expression pattern of ribosomal protein responsive to cold stress.** (TIFF 518 kb) Additional file 8:
**The expression pattern of 47 genes for pentatricopeptide repeat-containing protein and the expression pattern of the DEGs related to starch and sucrose metabolism.** (TIFF 256 kb) Additional file 9:
**Pathway related to starch and sucrose metabolism.** (TIFF 732 kb) Additional file 10:
**The change of transcript levels associated with lipid metabolism genes.** (TIFF 544 kb) Additional file 11:
**The expression pattern of the DEGs related to cell wall and cellulose.** (TIFF 611 kb) Additional file 12:
**The heat map of DEGs annotated as cytochrome P450s.** (TIFF 511 kb) Additional file 13:
**The heat map of DEGs annotated as ABC transporter.** (TIFF 526 kb) Additional file 14:
**The expression pattern of the DEGs related to phenylpropanoid metabolism pathway.** (TIFF 740 kb) Additional file 15:
**Pathway related to lignin synthesis.** (TIFF 362 kb) Additional file 16:
**The expression pattern of the DEGs related to flavonoid synthesis.** (TIFF 253 kb) Additional file 17:
**The expression pattern of the DEGs related to alkaloid metabolism.** (TIFF 314 kb) Additional file 18:
**The heat map of DEGs related to disease resistance protein.** (TIFF 787 kb) Additional file 19:
**The expression pattern of DEGs related to other resistance proteins.** (TIFF 605 kb) Additional file 20:
**Representative gel images of proteins from the control and treatment.** 2-DE was performed using 800 μg of total protein and 11 cm immobilized dry strips with linear pH gradients from 4 to 7. Gels were stained with CBB R-250. Arrow indicates proteins significantly changing in abundance in comparison with control (ANOVA, *p* < 0.05). Circle indicates proteins appeared after treatment. (TIFF 4732 kb) Additional file 21:
**The mean, SD and CV of 38 differential proteins with significant variance.** The protein No. is corresponding to the description in Fig. 6. (XLSX 22 kb) Additional file 22:
**PCs calculated from the 38 differential spot set (for cases and protein spots respectively).** (DOCX 25 kb) Additional file 23:
**PCA of differentially expressed proteins from respective time point treated samples.** **A** Projection of the cases. Gels for samples of control are represented as circles, for samples of 6 h cold treatment as squares, for samples of 12 h cold treatment as rhomboids, for samples of 24 h cold treatment as triangles, for samples of 48 h cold treatment as diamonds and for samples of 72 h cold treatment as ovals. **B** Projection of protein spots. Protein spots are clustered into five clusters according to PCA. Basically, proteins of ClusterIwere down-regulated during cold; proteins of cluster II, III, IV, V were up-regulated during cold. Proteins not clustered together indicated distinctive expression pattern from other clusters. (TIFF 221 kb) Additional file 24:
**The results of qPCR.** The correspondece protein spots of these gene ID were described in Additional file 25. The left axis represents the results of transcriptomics analysis while the right axis represents relative expression detected by qPCR. The orange column represented the proteomic results. qRT-PCR was performed with RNA isolated from leaves at 0 h, 2 h, 6 h, 12 h and 24 h. Actin gene of paper mulberry was used as an internal control. The final relative expression levels of genes were means of 3 replicates. The error bar represented three individual replicates. (TIFF 2365 kb) Additional file 25:
**The 34 differentially expressed protein spots and their correspondence unigenes in the RNA-seq data, as well as their primers designed for qPCR.** (XLSX 14 kb)
