# Supplementary figures and images for: The cold responsive mechanism of the paper mulberry: decreased photosynthesis capacity and increased starch accumulation
Source: BMC Genomics. 2015 Nov 5;16:898. doi: 10.1186/s12864-015-2047-6 (PMC4634900; doi:10.1186/s12864-015-2047-6)

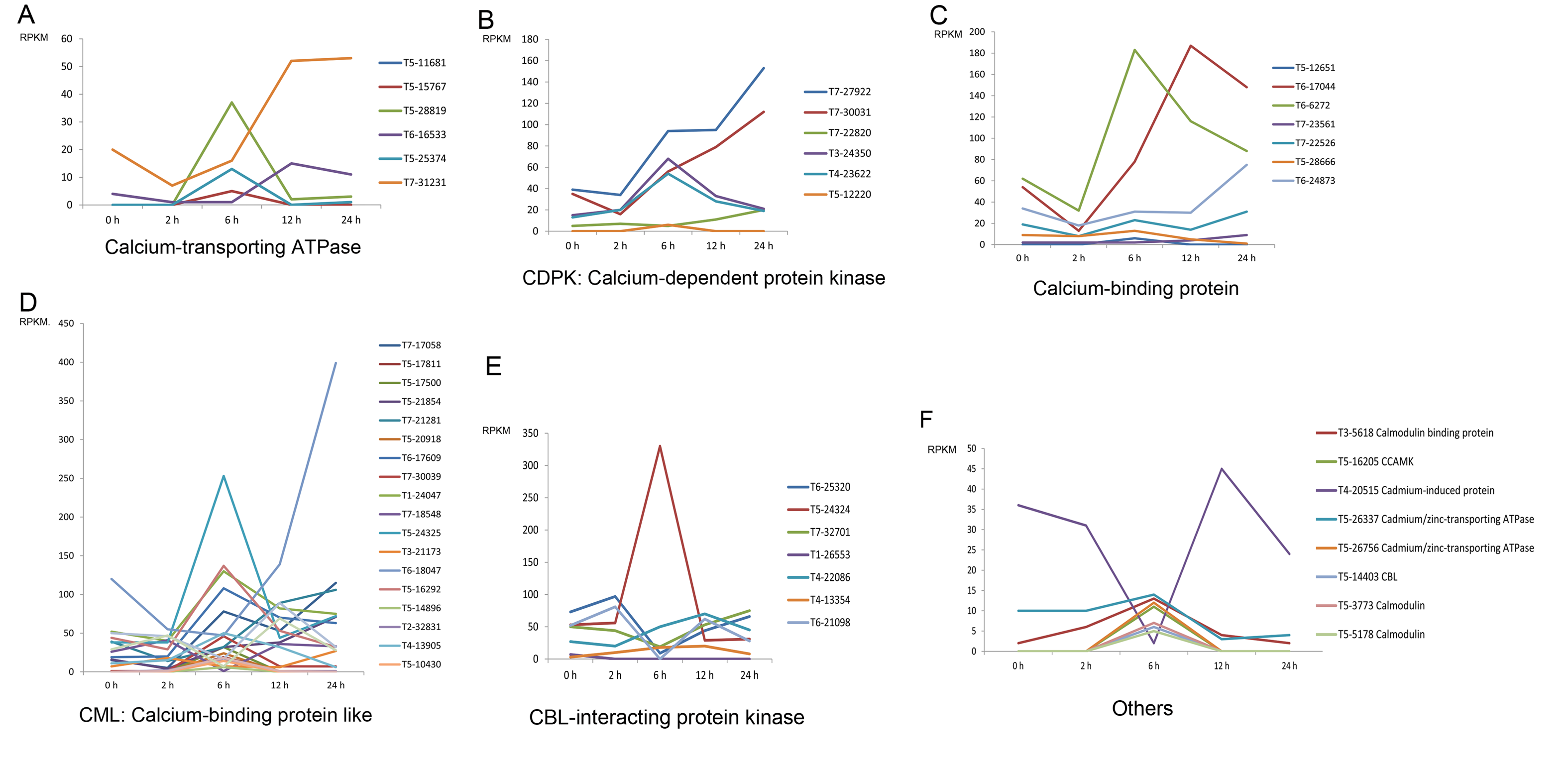

Supplement: Additional file 2: — The expression pattern of calcium signaling pathway related DEGs. (TIFF 804 kb) [file 12864_2015_2047_MOESM2_ESM.tiff]

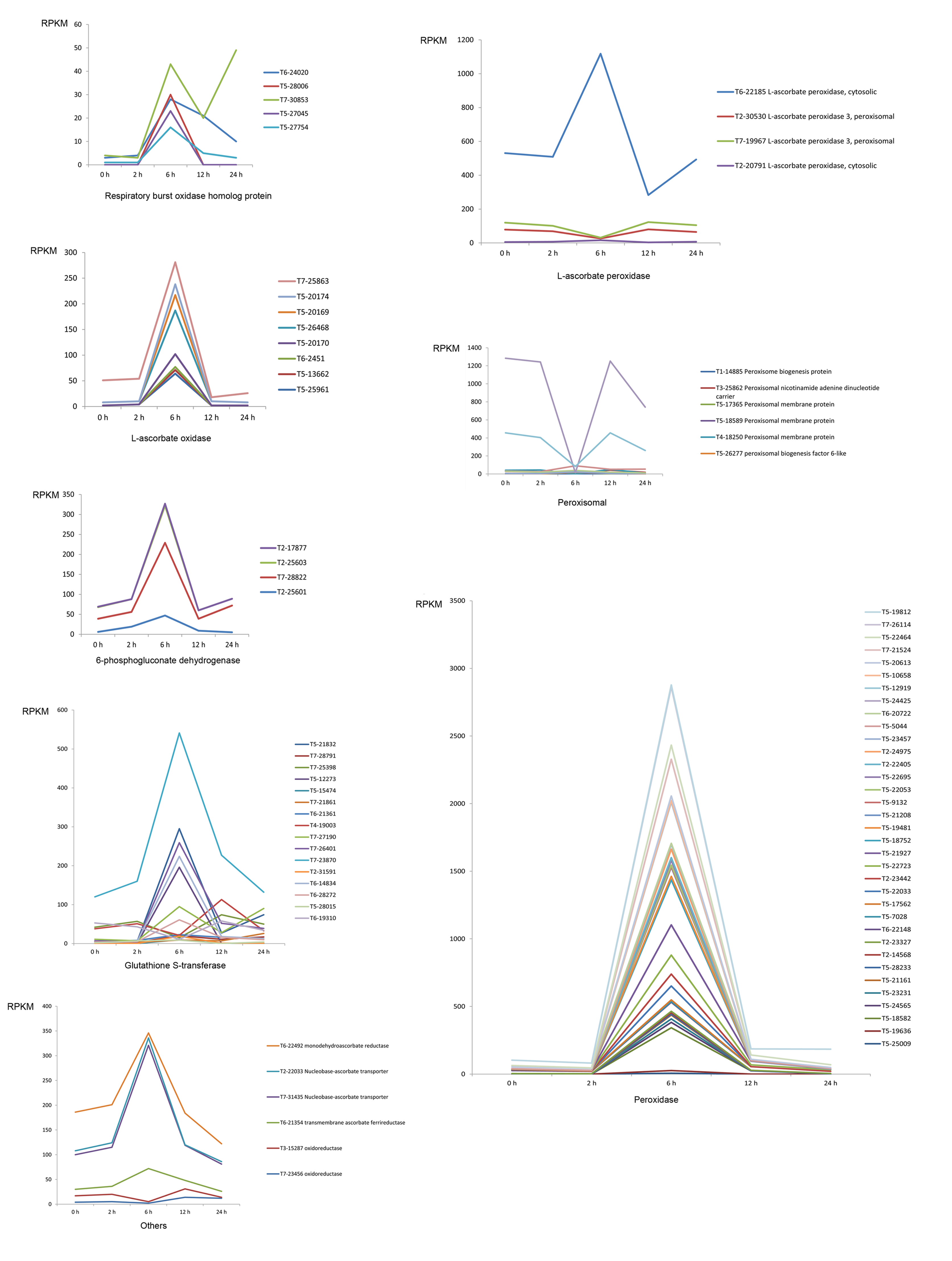

Supplement: Additional file 3: — The expression pattern of DEGs related ROS signaling pathway. (TIFF 2311 kb) [file 12864_2015_2047_MOESM3_ESM.tiff]

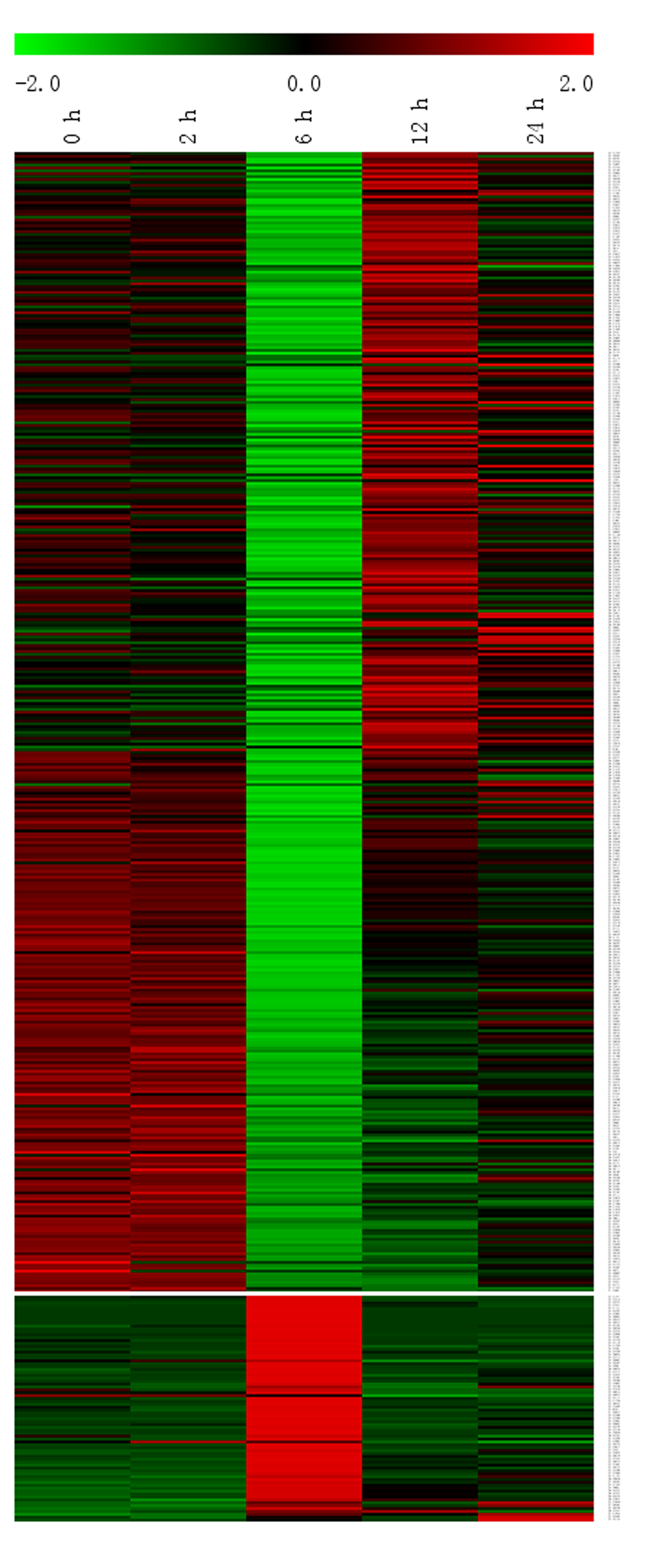

Supplement: Additional file 5: — Heat map of expression profiles of DEGs involved in the photosynthesis. According to their expression pattern under cold stress, the 472 DEGs were categorized in to two groups, including 393 and 79 DEGs, respectively. Red indicates high expression, black indicates intermediate expression, and green indicates low expression. (TIFF 634 kb) [file 12864_2015_2047_MOESM5_ESM.tiff]

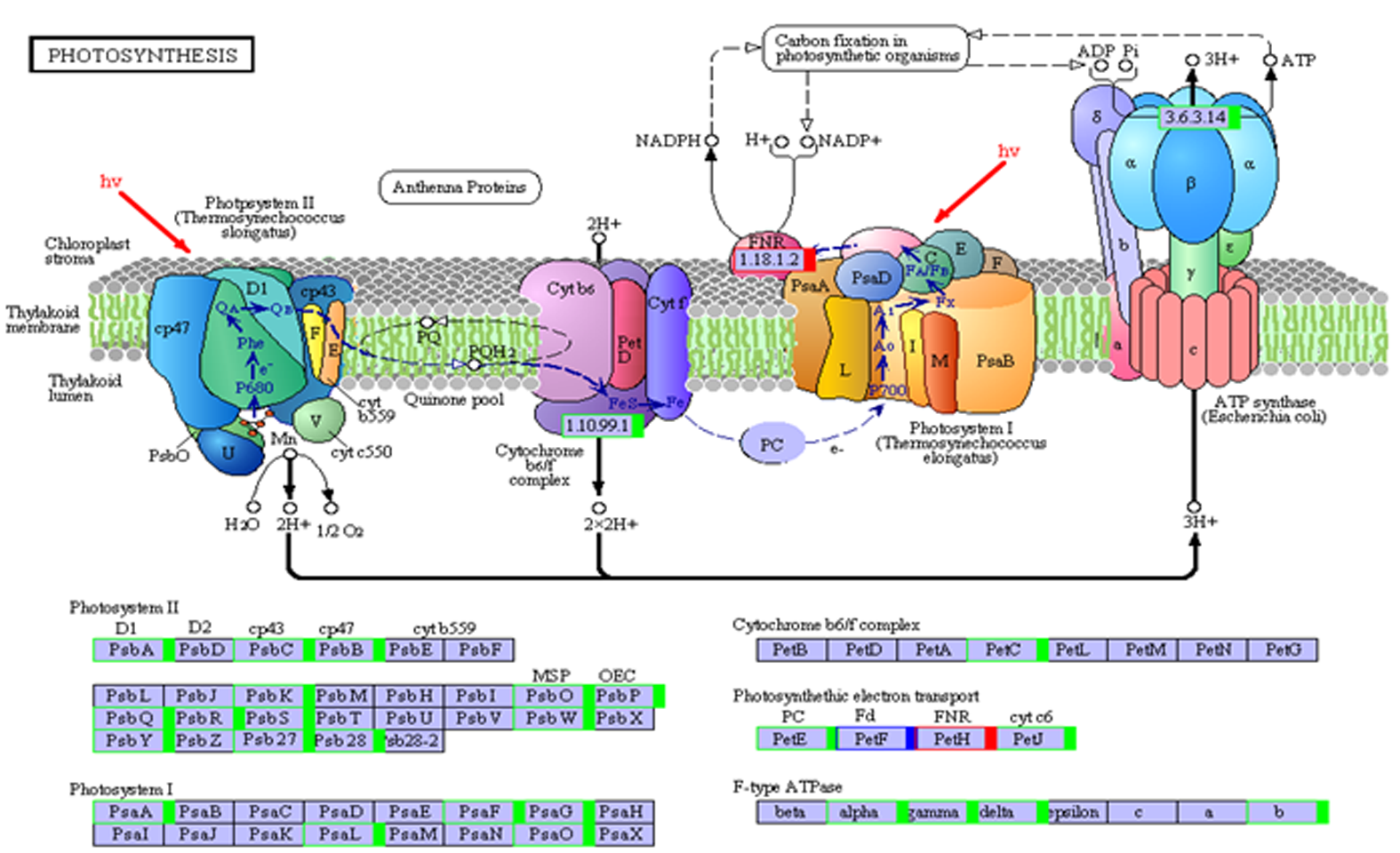

Supplement: Additional file 6: — The diagram illustrated for photosynthesis. (TIFF 1161 kb) [file 12864_2015_2047_MOESM6_ESM.tiff]

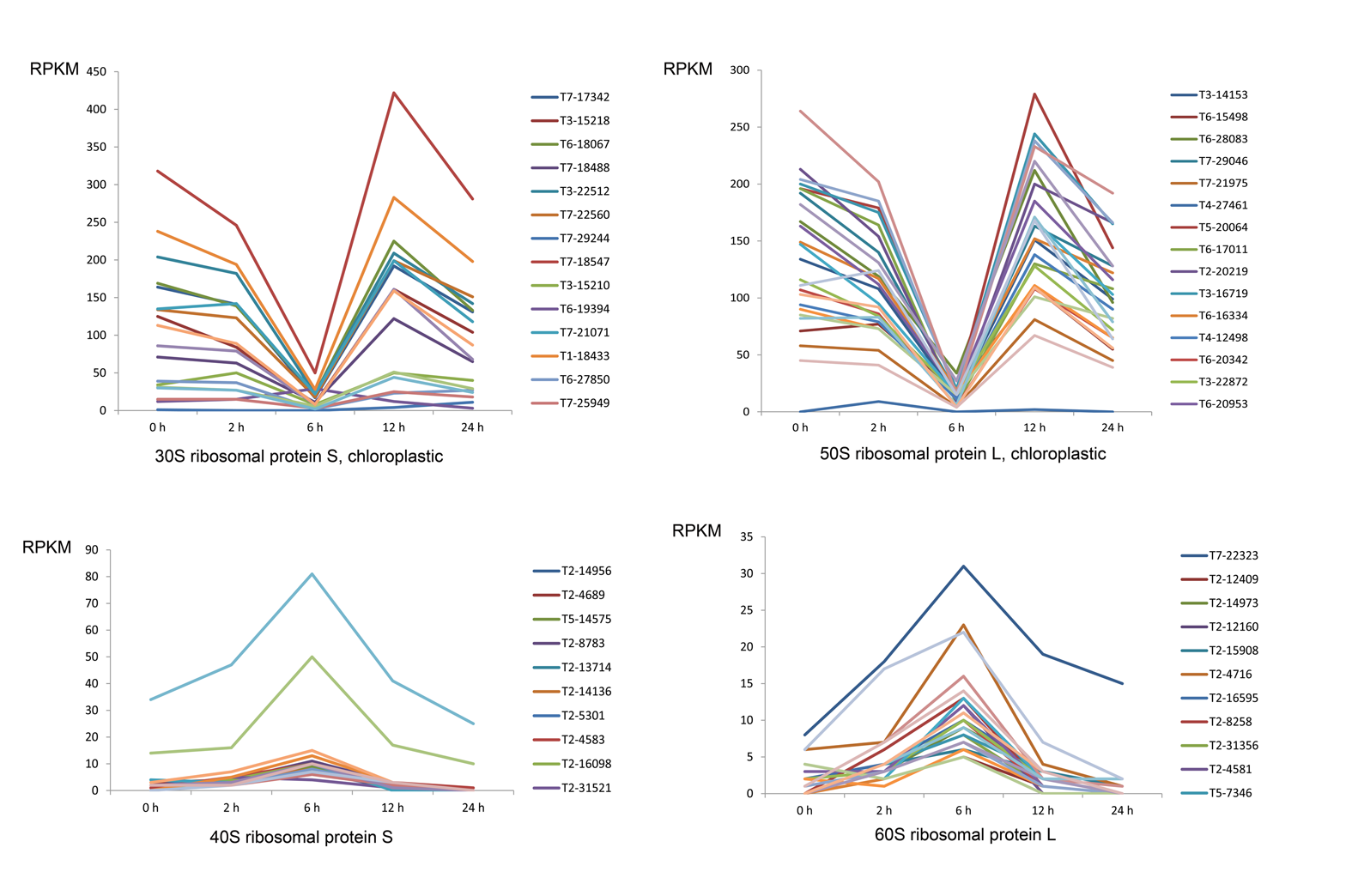

Supplement: Additional file 7: — The expression pattern of ribosomal protein responsive to cold stress. (TIFF 518 kb) [file 12864_2015_2047_MOESM7_ESM.tiff]

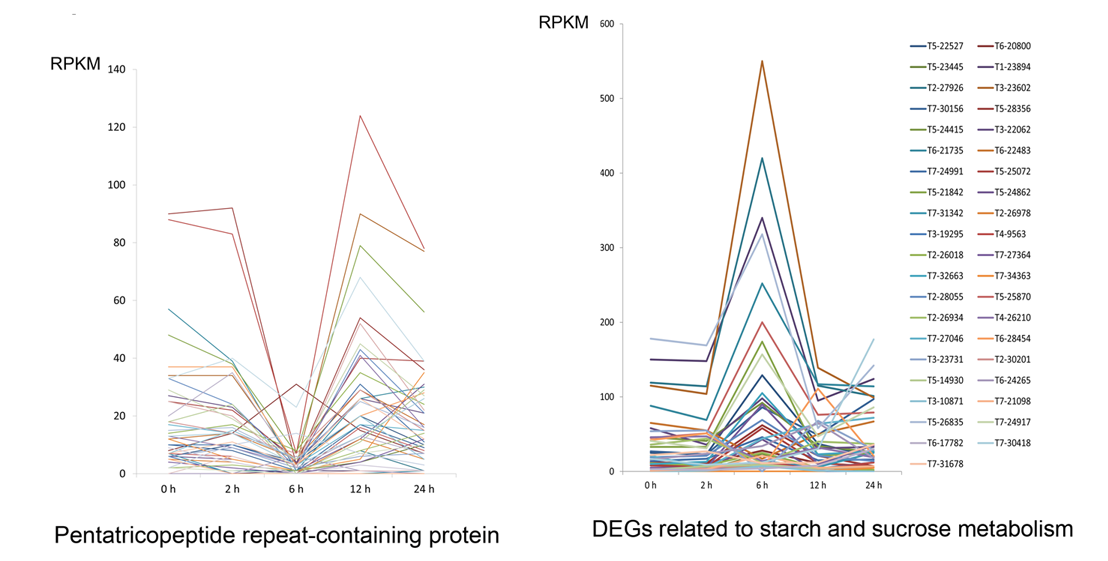

Supplement: Additional file 8: — The expression pattern of 47 genes for pentatricopeptide repeat-containing protein and the expression pattern of the DEGs related to starch and sucrose metabolism. (TIFF 256 kb) [file 12864_2015_2047_MOESM8_ESM.tiff]

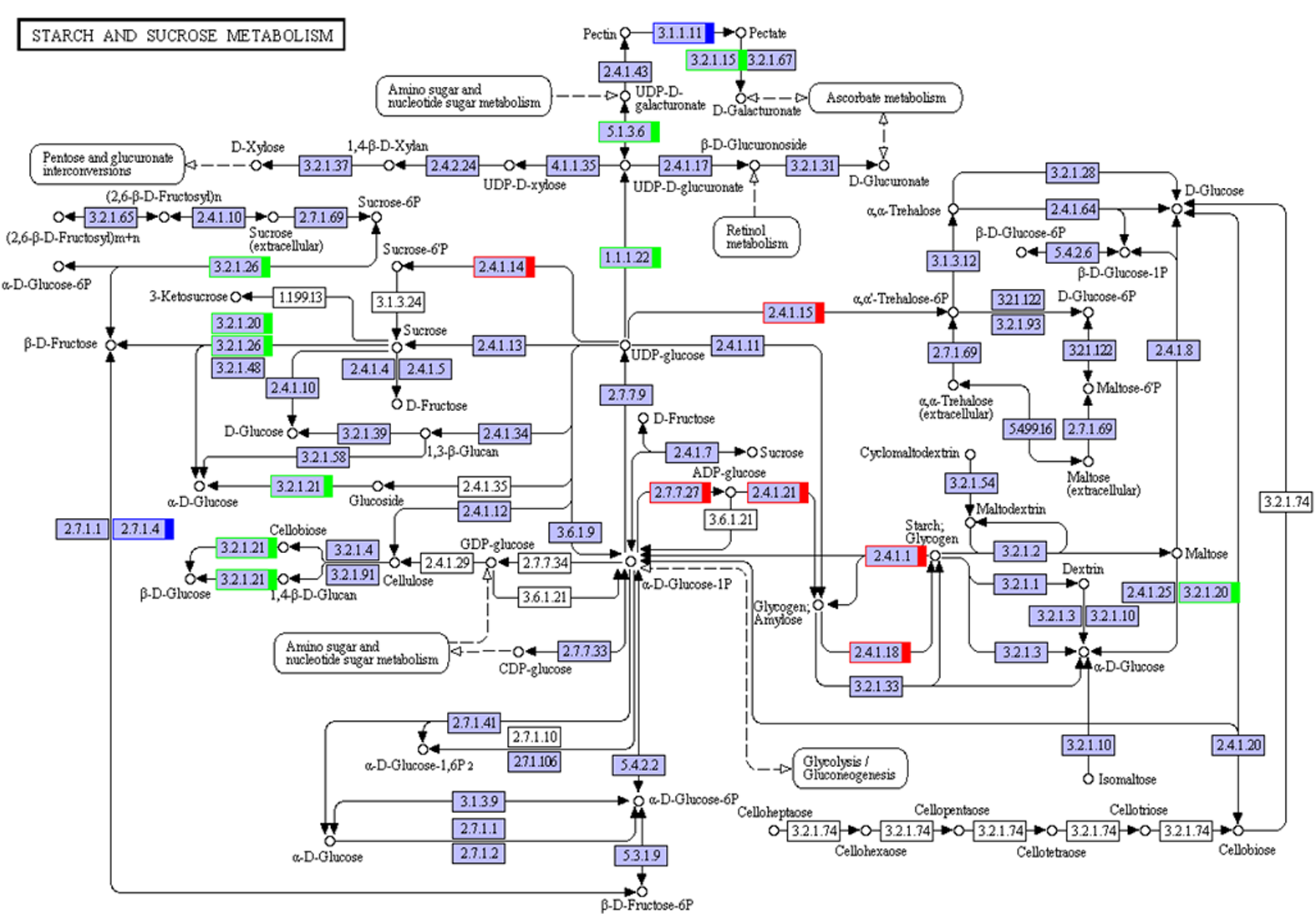

Supplement: Additional file 9: — Pathway related to starch and sucrose metabolism. (TIFF 732 kb) [file 12864_2015_2047_MOESM9_ESM.tiff]

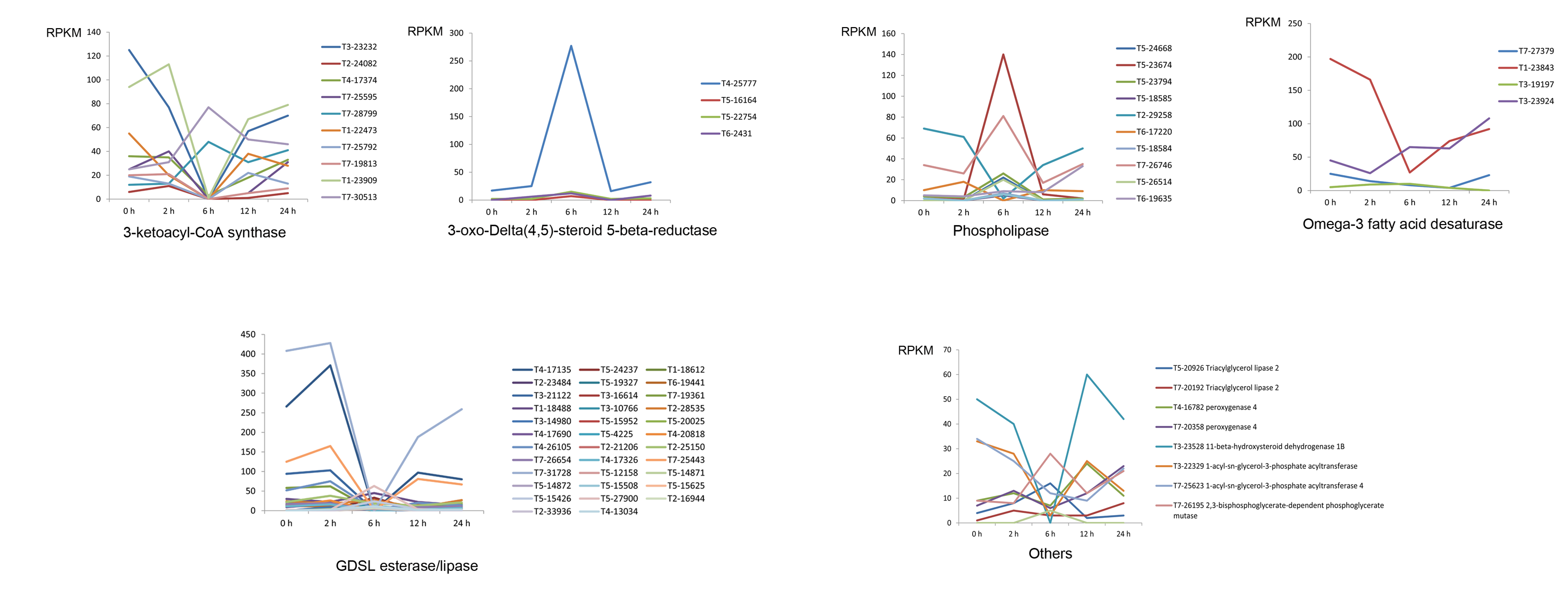

Supplement: Additional file 10: — The change of transcript levels associated with lipid metabolism genes. (TIFF 544 kb) [file 12864_2015_2047_MOESM10_ESM.tiff]

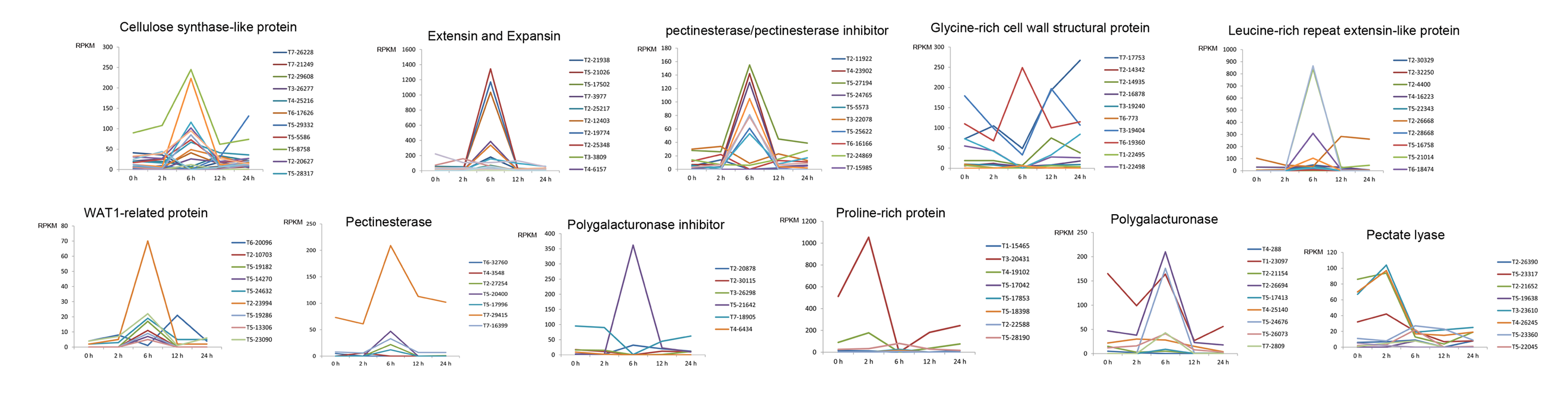

Supplement: Additional file 11: — The expression pattern of the DEGs related to cell wall and cellulose. (TIFF 611 kb) [file 12864_2015_2047_MOESM11_ESM.tiff]

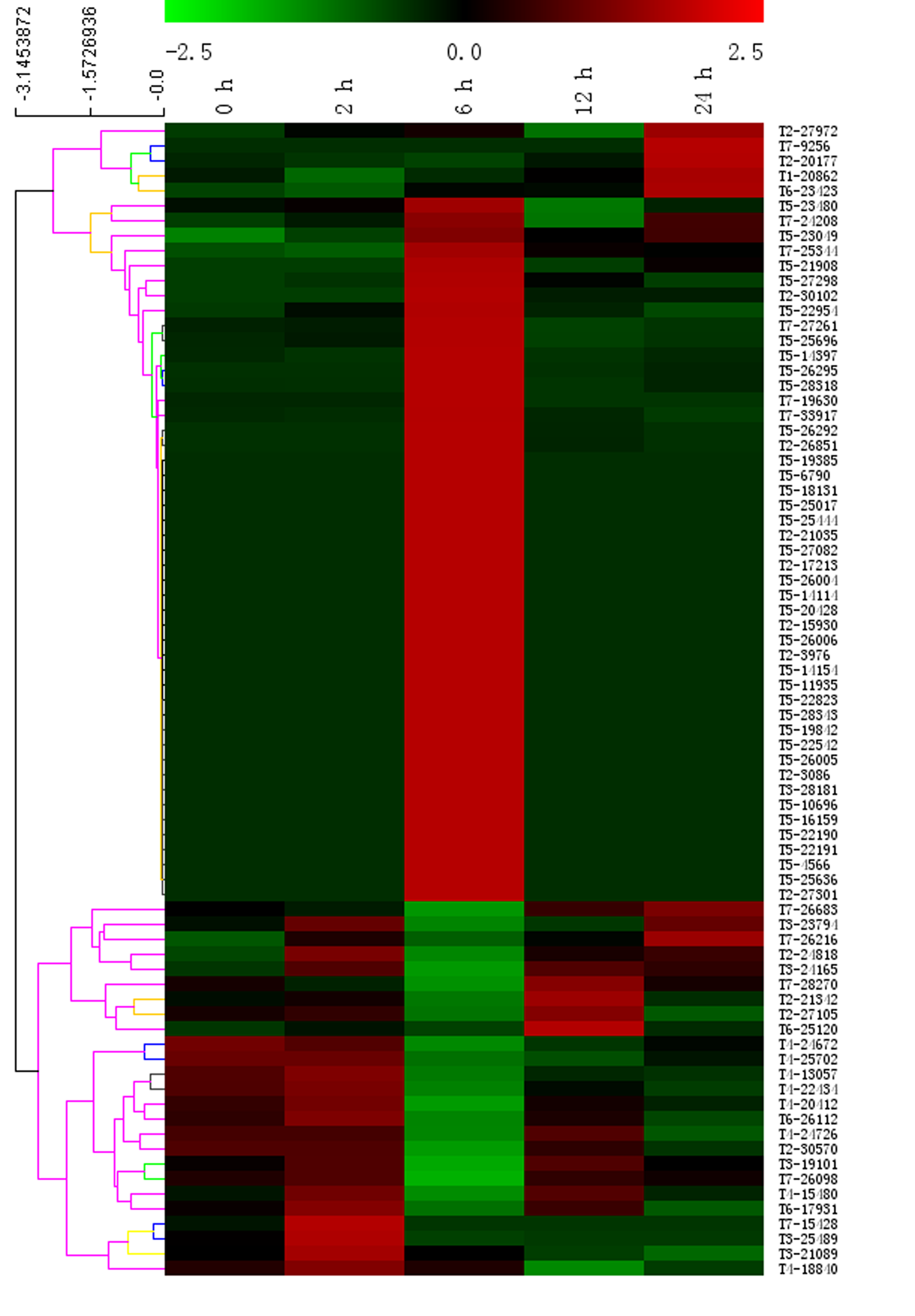

Supplement: Additional file 12: — The heat map of DEGs annotated as cytochrome P450s. (TIFF 511 kb) [file 12864_2015_2047_MOESM12_ESM.tiff]

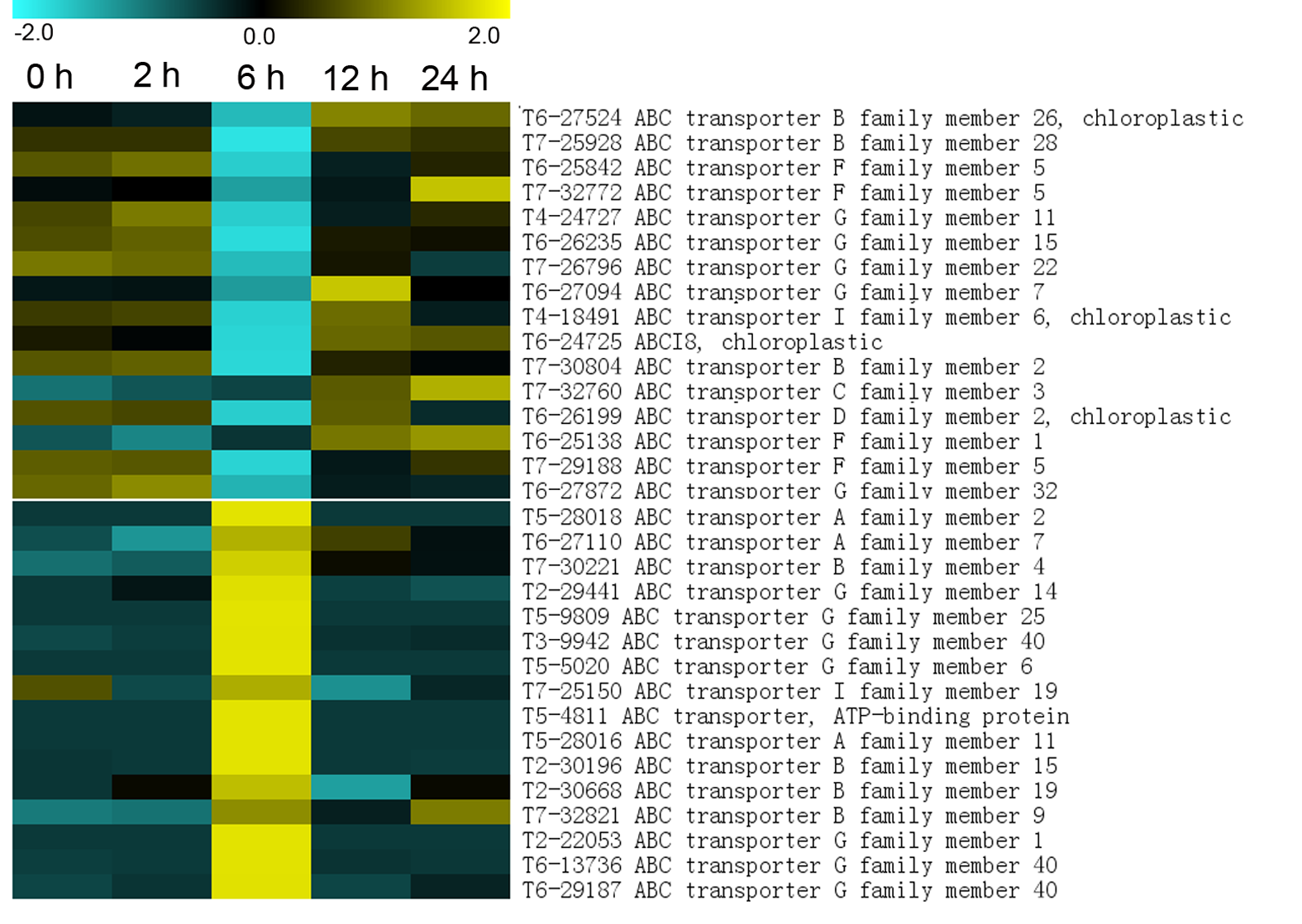

Supplement: Additional file 13: — The heat map of DEGs annotated as ABC transporter. (TIFF 526 kb) [file 12864_2015_2047_MOESM13_ESM.tiff]

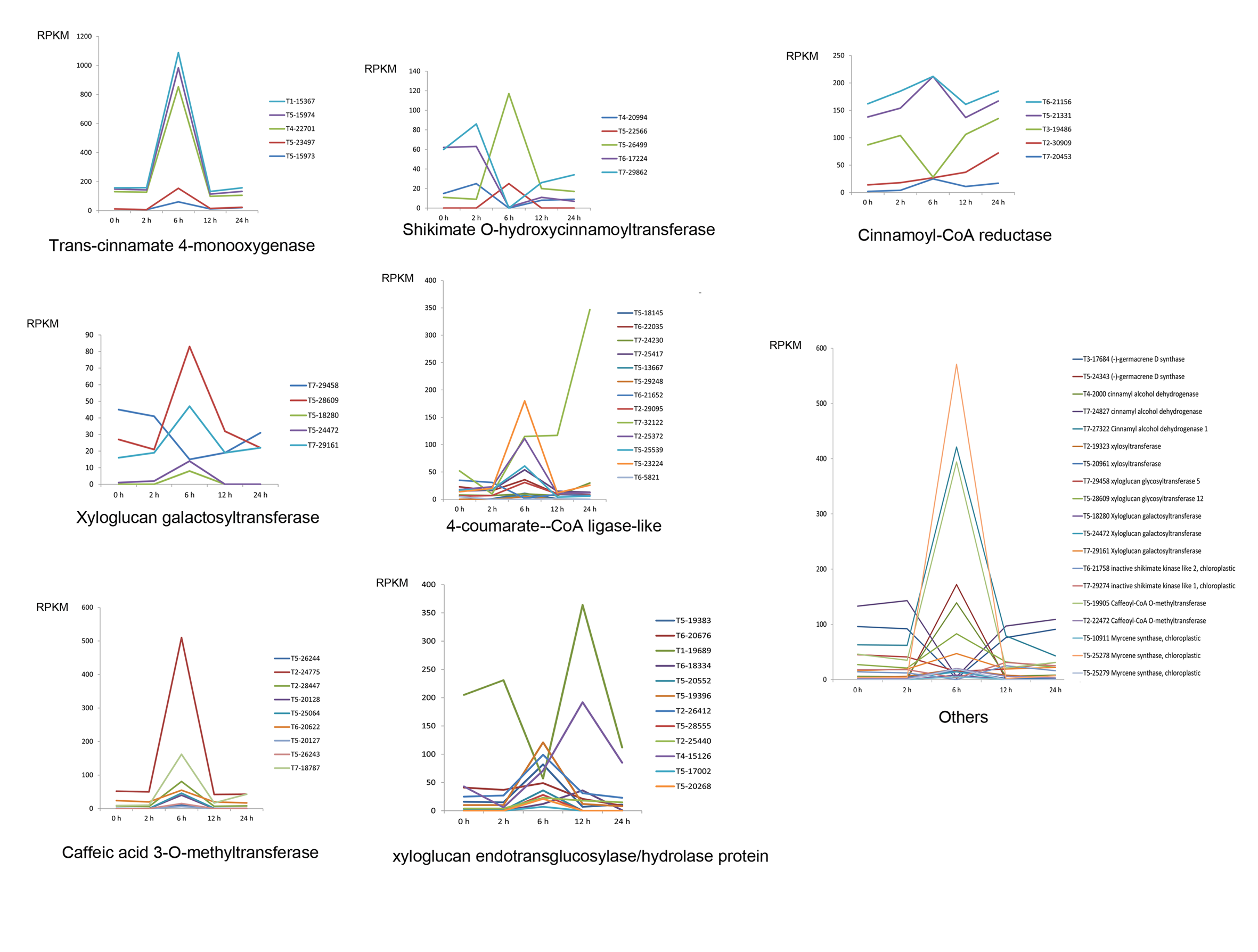

Supplement: Additional file 14: — The expression pattern of the DEGs related to phenylpropanoid metabolism pathway. (TIFF 740 kb) [file 12864_2015_2047_MOESM14_ESM.tiff]

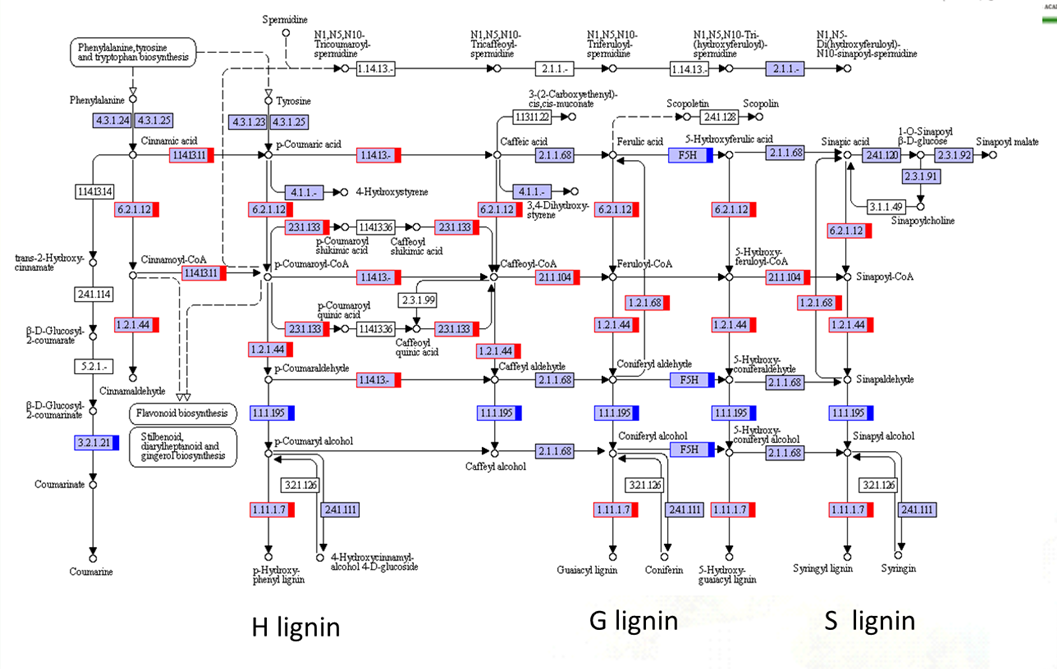

Supplement: Additional file 15: — Pathway related to lignin synthesis. (TIFF 362 kb) [file 12864_2015_2047_MOESM15_ESM.tiff]

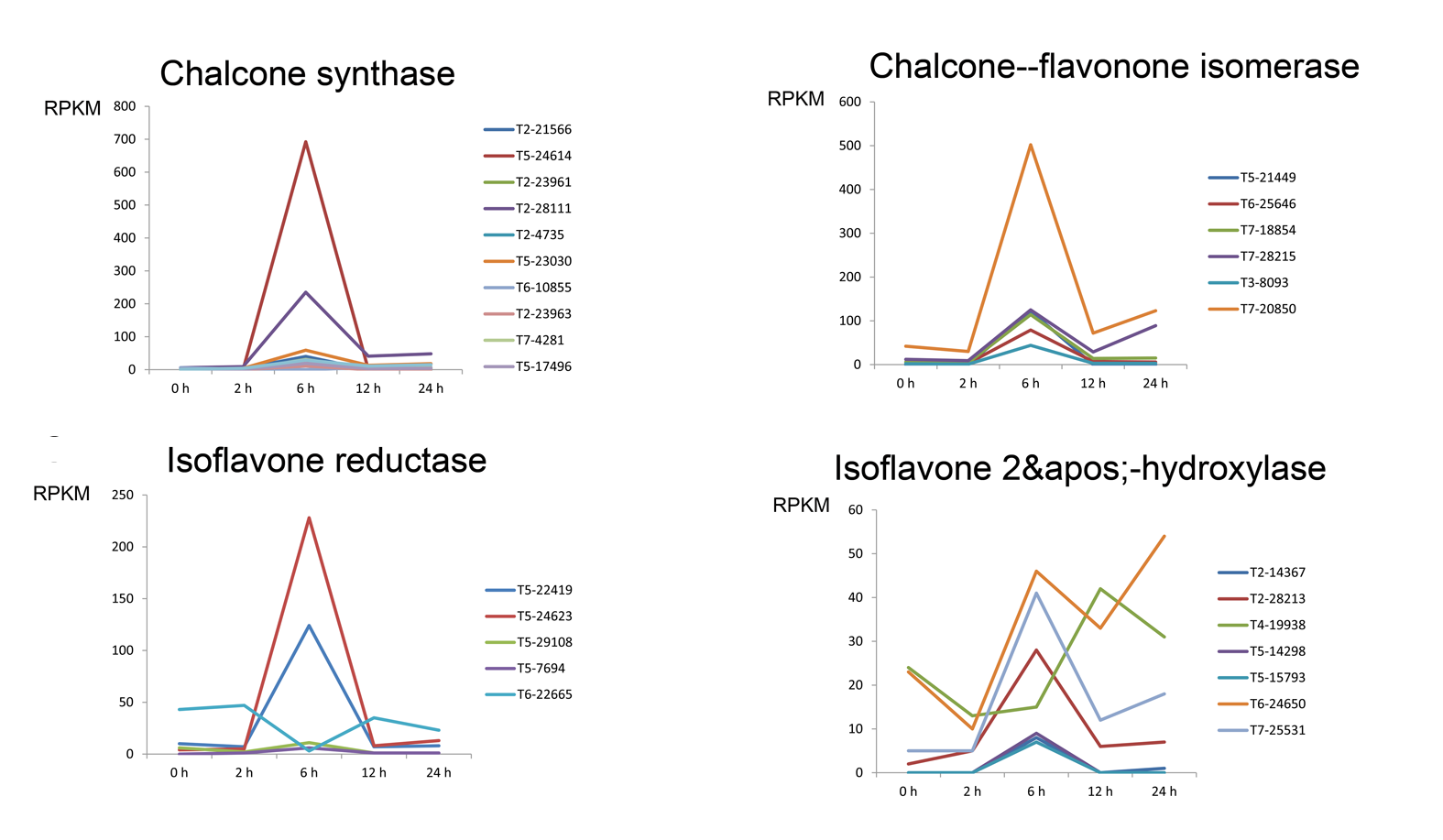

Supplement: Additional file 16: — The expression pattern of the DEGs related to flavonoid synthesis. (TIFF 253 kb) [file 12864_2015_2047_MOESM16_ESM.tiff]

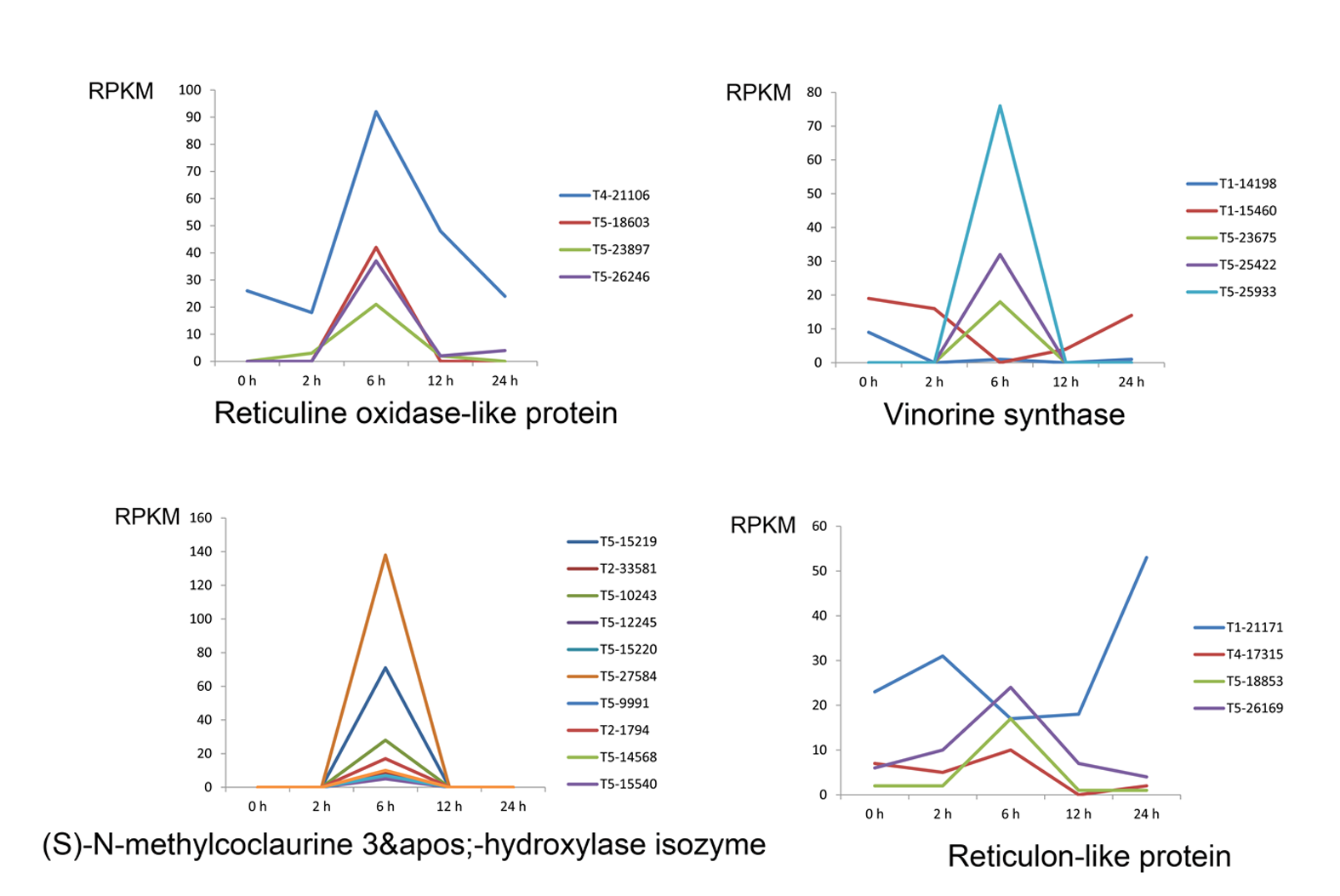

Supplement: Additional file 17: — The expression pattern of the DEGs related to alkaloid metabolism. (TIFF 314 kb) [file 12864_2015_2047_MOESM17_ESM.tiff]

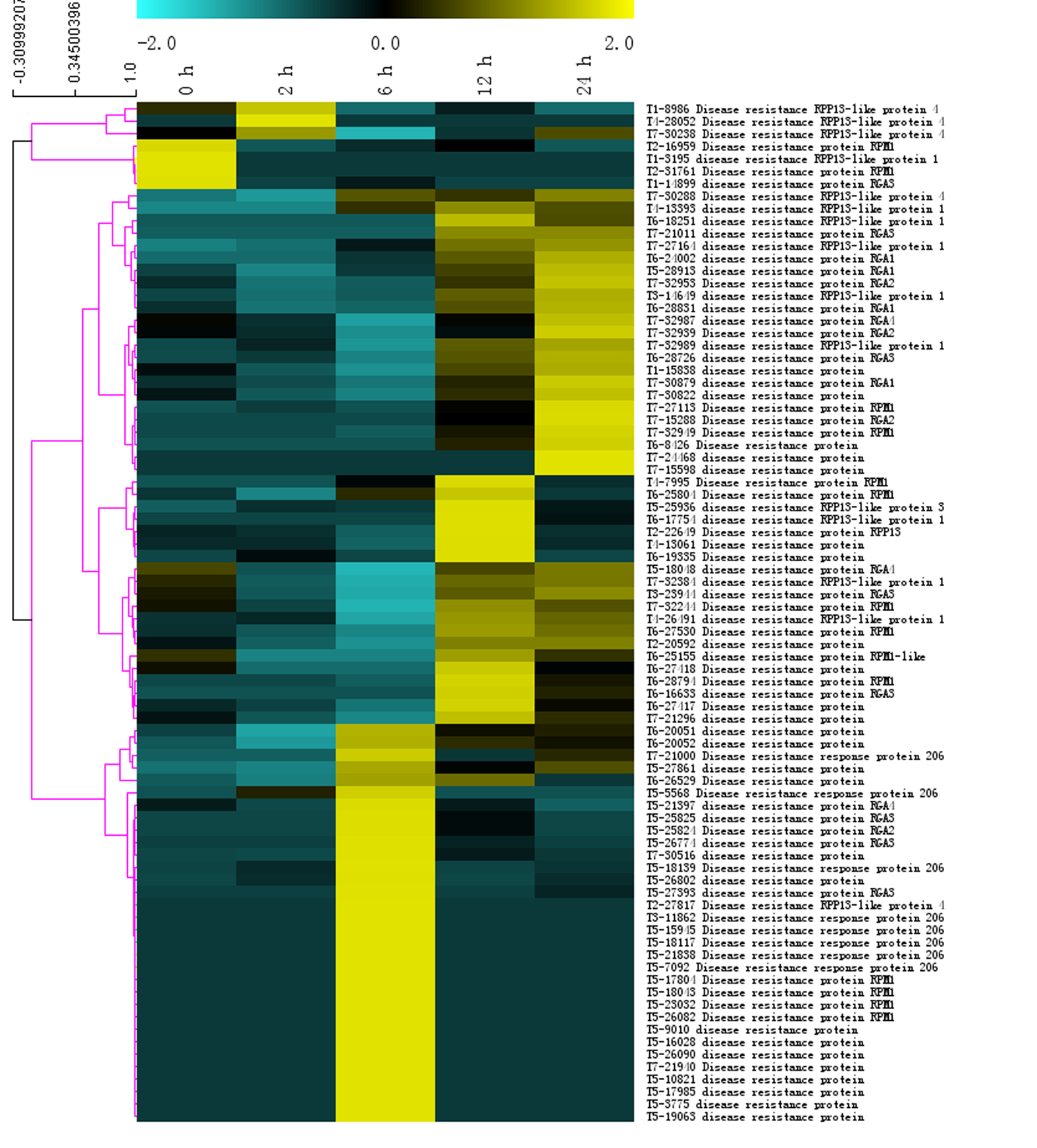

Supplement: Additional file 18: — The heat map of DEGs related to disease resistance protein. (TIFF 787 kb) [file 12864_2015_2047_MOESM18_ESM.tiff]

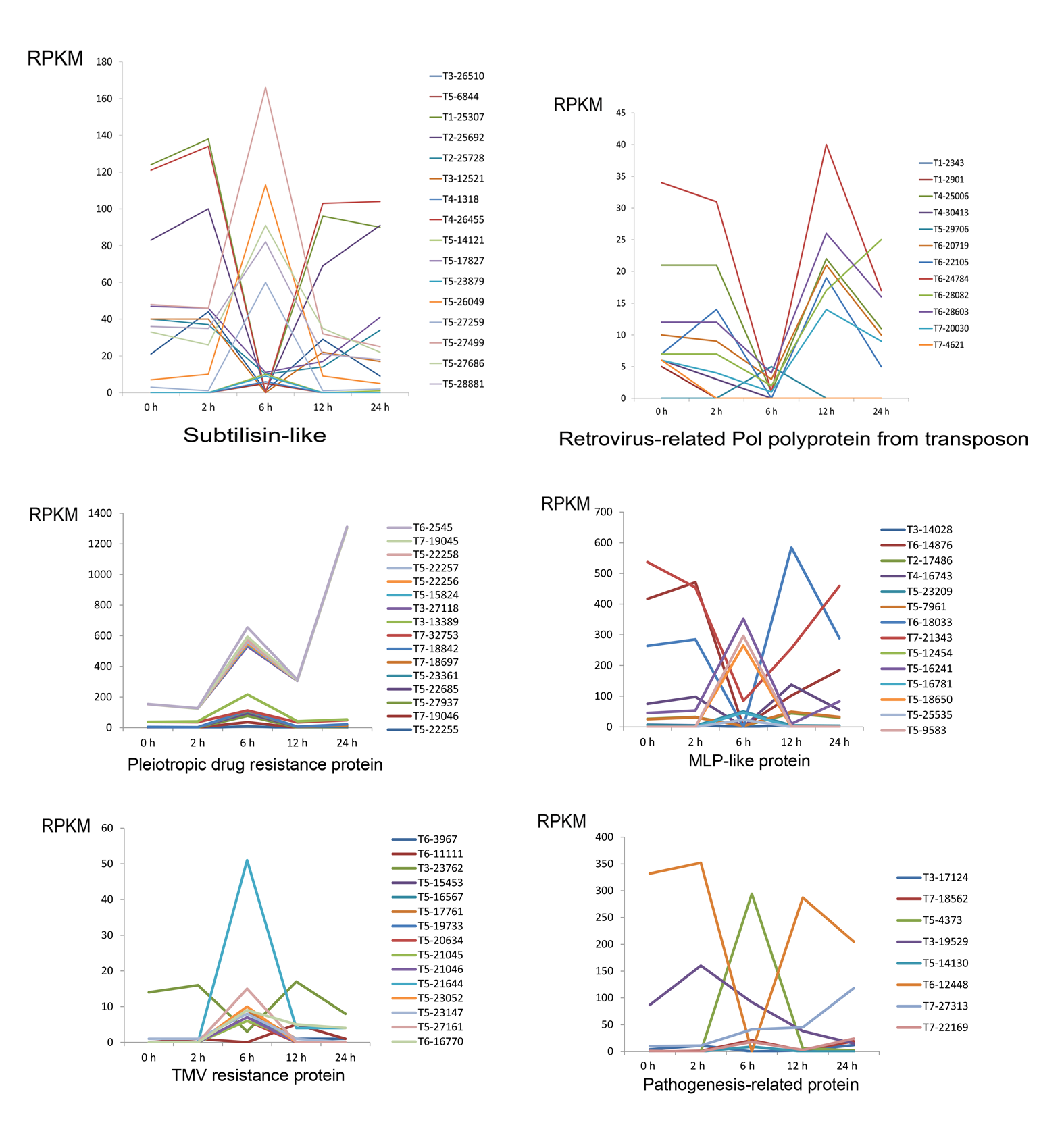

Supplement: Additional file 19: — The expression pattern of DEGs related to other resistance proteins. (TIFF 605 kb) [file 12864_2015_2047_MOESM19_ESM.tiff]

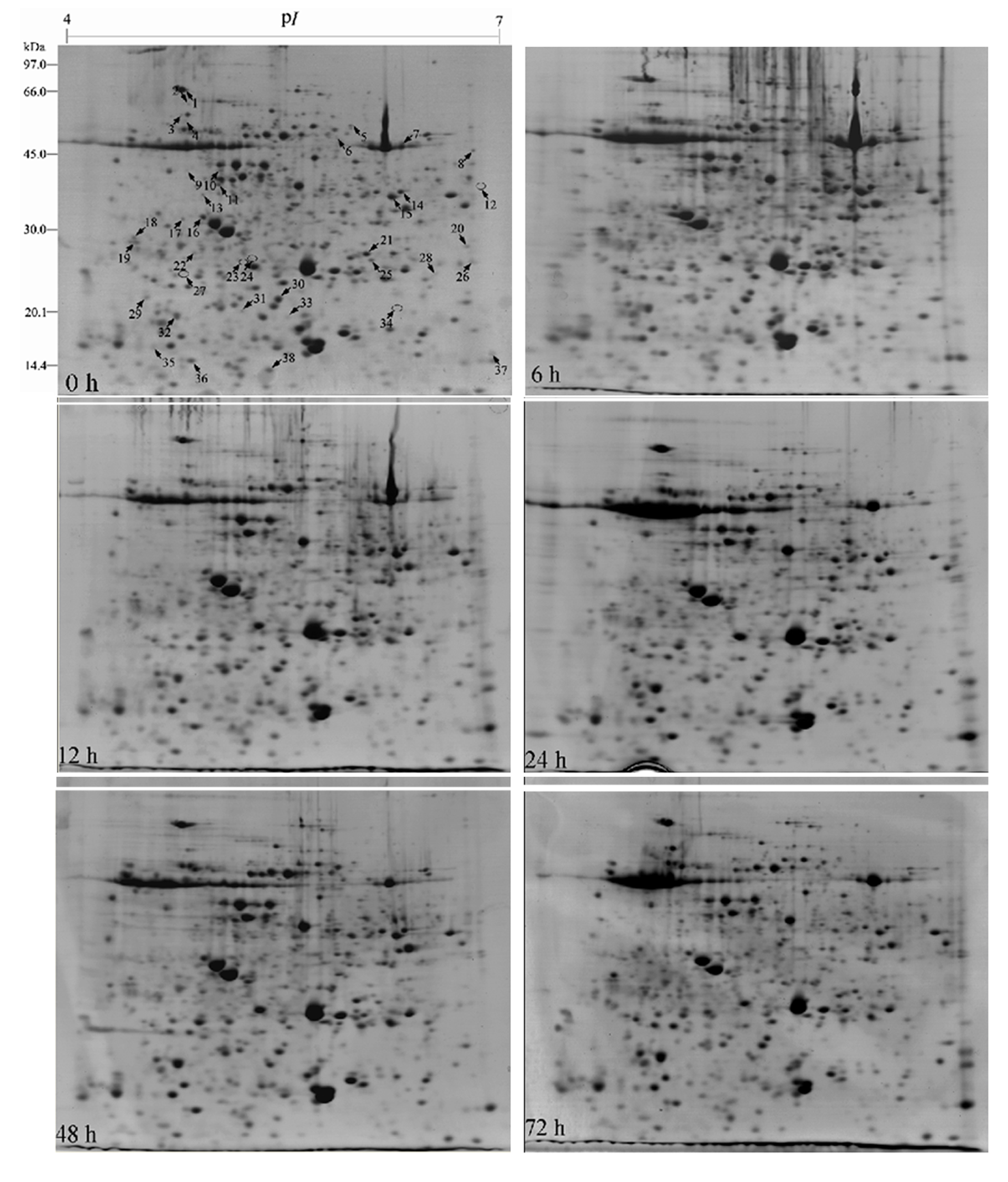

Supplement: Additional file 20: — Representative gel images of proteins from the control and treatment. 2-DE was performed using 800 μg of total protein and 11 cm immobilized dry strips with linear pH gradients from 4 to 7. Gels were stained with CBB R-250. Arrow indicates proteins significantly changing in abundance in comparison with control (ANOVA, p < 0.05). Circle indicates proteins appeared after treatment. (TIFF 4732 kb) [file 12864_2015_2047_MOESM20_ESM.tiff]

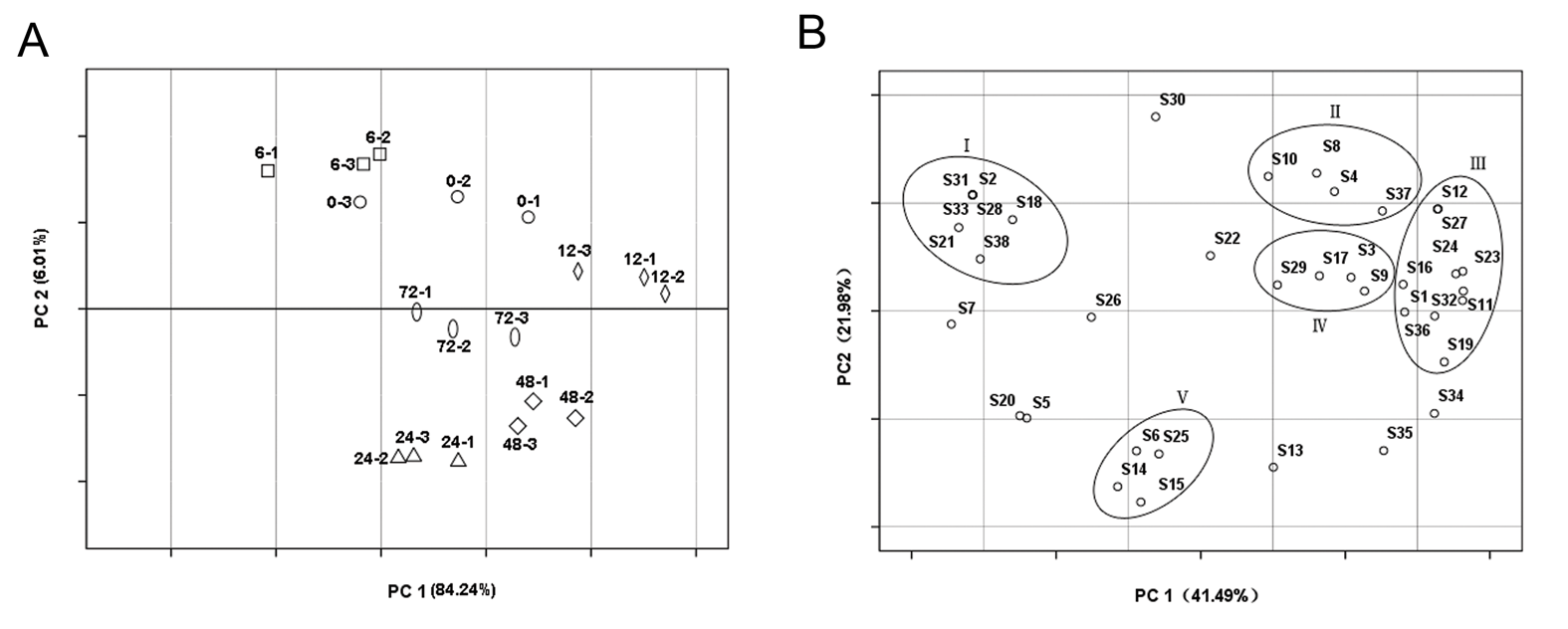

Supplement: Additional file 23: — PCA of differentially expressed proteins from respective time point treated samples. A Projection of the cases. Gels for samples of control are represented as circles, for samples of 6 h cold treatment as squares, for samples of 12 h cold treatment as rhomboids, for samples of 24 h cold treatment as triangles, for samples of 48 h cold treatment as diamonds and for samples of 72 h cold treatment as ovals. B Projection of protein spots. Protein spots are clustered into five clusters according to PCA. Basically, proteins of ClusterIwere down-regulated during cold; proteins of cluster II, III, IV, V were up-regulated during cold. Proteins not clustered together indicated distinctive expression pattern from other clusters. (TIFF 221 kb) [file 12864_2015_2047_MOESM23_ESM.tiff]

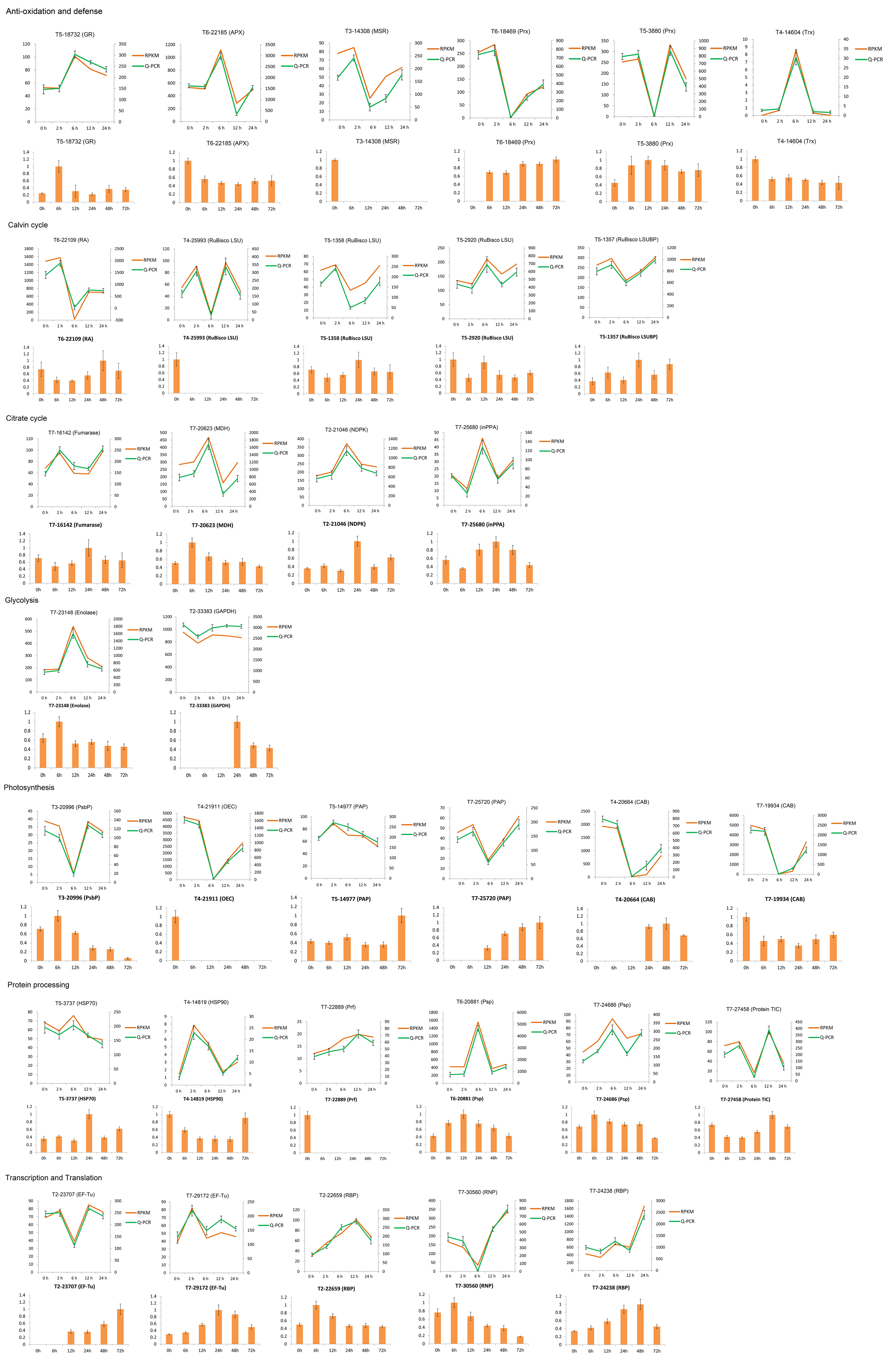

Supplement: Additional file 24: — The results of qPCR. The correspondece protein spots of these gene ID were described in Additional file 25. The left axis represents the results of transcriptomics analysis while the right axis represents relative expression detected by qPCR. The orange column represented the proteomic results. qRT-PCR was performed with RNA isolated from leaves at 0 h, 2 h, 6 h, 12 h and 24 h. Actin gene of paper mulberry was used as an internal control. The final relative expression levels of genes were means of 3 replicates. The error bar represented three individual replicates. (TIFF 2365 kb) [file 12864_2015_2047_MOESM24_ESM.tiff]
