# Supplementary material for: The cold responsive mechanism of the paper mulberry: decreased photosynthesis capacity and increased starch accumulation
Source: BMC Genomics. 2015 Nov 5;16:898. doi: 10.1186/s12864-015-2047-6 (PMC4634900; doi:10.1186/s12864-015-2047-6)
Supplement: Additional file 22: — PCs calculated from the 38 differential spot set (for cases and protein spots respectively). (DOCX 25 kb) [file 12864_2015_2047_MOESM22_ESM.docx]

**Supporting Information 23** PCs calculated from the 38 differential spot set (for cases and protein spots respectively)

| **Total Variance Explained** | | | | | | |
| --- | --- | --- | --- | --- | --- | --- |
| Component | Initial Eigenvalues | | | Extraction Sums of Squared Loadings | | |
|  | Total | % of Variance | Cumulative % | Total | % of Variance | Cumulative % |
| 1 | 15.164 | 84.244 | 84.244 | 15.164 | 84.244 | 84.244 |
| 2 | 1.083 | 6.014 | 90.258 | 1.083 | 6.014 | 90.258 |
| 3 | .601 | 3.340 | 93.598 |  |  |  |
| 4 | .406 | 2.254 | 95.852 |  |  |  |
| 5 | .206 | 1.143 | 96.995 |  |  |  |
| 6 | .144 | .799 | 97.793 |  |  |  |
| 7 | .111 | .618 | 98.411 |  |  |  |
| 8 | .064 | .358 | 98.769 |  |  |  |
| 9 | .059 | .330 | 99.099 |  |  |  |
| 10 | .043 | .241 | 99.340 |  |  |  |
| 11 | .033 | .181 | 99.520 |  |  |  |
| 12 | .031 | .170 | 99.690 |  |  |  |
| 13 | .021 | .114 | 99.804 |  |  |  |
| 14 | .012 | .069 | 99.873 |  |  |  |
| 15 | .009 | .050 | 99.923 |  |  |  |
| 16 | .007 | .038 | 99.961 |  |  |  |
| 17 | .005 | .027 | 99.988 |  |  |  |
| 18 | .002 | .012 | 100.000 |  |  |  |
| Extraction Method: Principal Component Analysis. | | | | | | |

| **Total Variance Explained** | | | | | | |
| --- | --- | --- | --- | --- | --- | --- |
| Component | Initial Eigenvalues | | | Extraction Sums of Squared Loadings | | |
|  | Total | % of Variance | Cumulative % | Total | % of Variance | Cumulative % |
| 1 | 15.768 | 41.494 | 41.494 | 15.768 | 41.494 | 41.494 |
| 2 | 8.354 | 21.984 | 63.478 | 8.354 | 21.984 | 63.478 |
| 3 | 4.752 | 12.505 | 75.983 | 4.752 | 12.505 | 75.983 |
| 4 | 2.900 | 7.633 | 83.615 | 2.900 | 7.633 | 83.615 |
| 5 | 1.669 | 4.393 | 88.008 | 1.669 | 4.393 | 88.008 |
| 6 | 1.229 | 3.234 | 91.242 | 1.229 | 3.234 | 91.242 |
| 7 | .702 | 1.849 | 93.090 |  |  |  |
| 8 | .566 | 1.488 | 94.579 |  |  |  |
| 9 | .539 | 1.418 | 95.997 |  |  |  |
| 10 | .379 | .997 | 96.994 |  |  |  |
| 11 | .361 | .949 | 97.944 |  |  |  |
| 12 | .253 | .667 | 98.611 |  |  |  |
| 13 | .176 | .463 | 99.074 |  |  |  |
| 14 | .145 | .382 | 99.456 |  |  |  |
| 15 | .101 | .266 | 99.722 |  |  |  |
| 16 | .066 | .173 | 99.895 |  |  |  |
| 17 | .040 | .105 | 100.000 |  |  |  |
| 18 | 2.402E-15 | 6.321E-15 | 100.000 |  |  |  |
| 19 | 9.009E-16 | 2.371E-15 | 100.000 |  |  |  |
| 20 | 7.363E-16 | 1.938E-15 | 100.000 |  |  |  |
| 21 | 5.439E-16 | 1.431E-15 | 100.000 |  |  |  |
| 22 | 4.710E-16 | 1.239E-15 | 100.000 |  |  |  |
| 23 | 4.417E-16 | 1.162E-15 | 100.000 |  |  |  |
| 24 | 3.513E-16 | 9.245E-16 | 100.000 |  |  |  |
| 25 | 2.222E-16 | 5.846E-16 | 100.000 |  |  |  |
| 26 | 1.863E-16 | 4.902E-16 | 100.000 |  |  |  |
| 27 | 1.755E-16 | 4.618E-16 | 100.000 |  |  |  |
| 28 | 1.037E-16 | 2.728E-16 | 100.000 |  |  |  |
| 29 | -3.033E-17 | -7.982E-17 | 100.000 |  |  |  |
| 30 | -8.384E-17 | -2.206E-16 | 100.000 |  |  |  |
| 31 | -1.571E-16 | -4.135E-16 | 100.000 |  |  |  |
| 32 | -2.035E-16 | -5.355E-16 | 100.000 |  |  |  |
| 33 | -2.687E-16 | -7.070E-16 | 100.000 |  |  |  |
| 34 | -3.179E-16 | -8.365E-16 | 100.000 |  |  |  |
| 35 | -4.028E-16 | -1.060E-15 | 100.000 |  |  |  |
| 36 | -4.376E-16 | -1.152E-15 | 100.000 |  |  |  |
| 37 | -6.838E-16 | -1.799E-15 | 100.000 |  |  |  |
| 38 | -9.893E-16 | -2.603E-15 | 100.000 |  |  |  |
| Extraction Method: Principal Component Analysis. | | | | | | |
